# Application of transformer attention mechanism-based multimodal deep learning model in the diagnosis of papillary thyroid carcinoma

**DOI:** 10.1186/s12880-026-02530-w

**Published:** 2026-06-27

**Authors:** Min Xu, Xufeng Zhu, Zhaoyang Lu, Weihua Xu, Chunming Shen, Jing Yang

**Affiliations:** 1 Department of Ultrasound, Suzhou Ninth People’s Hospital, Suzhou, Jiangsu Province China; 2 Department of Ultrasound, The Affiliated Jiangsu Shengze Hospital of Nanjing Medical University, Suzhou, Jiangsu Province China; 3 Oncology Center, The Affiliated Jiangsu Shengze Hospital of Nanjing Medical University, Suzhou, Jiangsu Province China

**Keywords:** Papillary thyroid carcinoma, Deep learning, Transformer, Multimodal fusion, End-to-end learning, Ablation experiment

## Abstract

**Objective:**

A Transformer-based multimodal deep learning model was developed to enhance ultrasound imaging diagnosis of PTC and benign nodules.

**Methods:**

This study included 491 thyroid nodule patients from two centers, with 406 from the Suzhou Ninth People’s Hospital divided into training (*n* = 284) and validation (*n* = 122) sets, and 85 from Jiangsu Shengze Hospital as an external test set. The dataset comprised 232 benign nodules and 259 papillary thyroid carcinoma cases. A comparison of 34 deep learning architectures and four traditional models was conducted, leading to the proposal of an Efficientnetv2scbamTrans fusion model. Five ablation experiments assessed module contributions, while SHAP and Grad-CAM were used for interpretability, with energy concentration, peak center distance, and spatial IoU as performance indicators.

**Results:**

DenseNet201 excelled on the internal validation set but showed overfitting with an AUC of 0.702 on the external test set. The unimodal imaging baseline had an AUC of 0.782, which improved to 0.811 with clinical features, reducing overfitting risk. Directly fusing radiomics features yielded no improvement, maintaining an AUC of 0.751. The proposed Efficientnetv2scbamTrans model reached an AUC of 0.985 on the internal validation set. More importantly, on the independent external test set (Jiangsu Shengze Hospital), the model achieved an AUC of 0.986 (95% CI: 0.967–1.000), with 96.4% sensitivity and 96.7% specificity. On this external test set, our proposed model significantly outperformed the traditional DLR model by an AUC margin of 0.199 (*p* < 0.001). SHAP analysis indicated clinical features altered radiomics decision weights, and Grad-CAM showed an increase in spatial IoU from 0.097 to 0.349, enhancing visual localization.

**Conclusion:**

The study developed a multimodal deep learning model using thyroid ultrasound, improving diagnostics with interaction and attention mechanisms via CBAM and Transformer.

**Supplementary Information:**

The online version contains supplementary material available at https://doi.org/10.1186/s12880-026-02530-w.

## Background

Thyroid nodules, as a common endocrine system disease, have an increasing incidence rate year by year, particularly significantly higher in the female population. The prevalence of thyroid nodules in the general population ranges from 20% to 76% [1]. Most nodules are benign. However, about 7% to 15% may be malignant, making early and accurate diagnosis particularly important [2]. Papillary thyroid carcinoma (PTC) is the most common type of thyroid cancer, accounting for about 80% to 90% of all cases [3]. However, PTC and benign nodules with fibrosis appear very similar in ultrasound imaging. Both show features such as low echogenicity, irregular borders, and microcalcifications. This similarity causes difficulties in preoperative diagnosis and results in a high false-positive rate of 30% to 50% [4]. Traditional ultrasound diagnosis relies on doctors’ subjective judgment. Consistency among diagnosticians is poor (Kappa = 0.4–0.6) [5], which leads to many unnecessary fine-needle aspiration biopsies and surgical interventions. Research on thyroid nodules has shown that radiomics features improve diagnostic accuracy and are closely related to patient prognosis [6, 7]. Studies show that analyzing imaging features better determines the nature of the nodule, providing a more reliable basis for treatment planning [8]. However, current research lacks comprehensive integration of deep learning and traditional radiomics. The use of ultrasound imaging classification systems (such as TIRADS) can effectively reduce the number of unnecessary fine-needle aspiration biopsies and improve the detection rate of malignant nodules [9, 10]. Although fine-needle aspiration biopsy is the gold standard, its invasiveness and false-negative rates limit its widespread use among all suspected patients [11].

In recent years, deep learning has made significant progress in the field of medical image analysis [12]. For the diagnosis of thyroid nodules, some studies have attempted to use convolutional neural networks (CNN) for benign-malignant classification [13–15]. However, existing research has some issues. Traditional multi-modal fusion methods typically follow a “two-stage paradigm”. First, they extract deep learning features and handcrafted features (such as radiomics). Then, machine learning classifiers (such as random forests or support vector machines) are used for fusion classification [16, 17]. This approach separates feature extraction from classification, hindering global joint optimization and complicating the modeling of complex non-linear interactions and conditional dependencies between features. In contrast, end-to-end learning employs a unified deep network architecture that directly maps the original input to the final output. Gradients backpropagate from the loss function to all parameters, enabling global optimization [18]. The convolutional block attention module (CBAM) adaptively enhances key regions and feature channels in diagnosis through channel attention and spatial attention [19]. However, the exact impact of these mechanisms on thyroid ultrasound tasks remains unquantified.

This study aims to systematically compare the performance of deep learning models based on natural image pre-training in thyroid ultrasound tasks. It also validates performance improvements through multi-modal fusion and structural optimization. Furthermore, it verifies negative transfer effects and compares the performance differences between traditional two-stage fusion methods and end-to-end multi-modal learning. Finally, carefully designed ablation experiments quantitatively evaluate the independent contributions of CBAM attention mechanisms, multi-modal fusion modules based on Transformer, and multi-modal collaborative mechanisms.

## Materials and methods

### Patient cohort and data partitioning

**Fig. 1:**
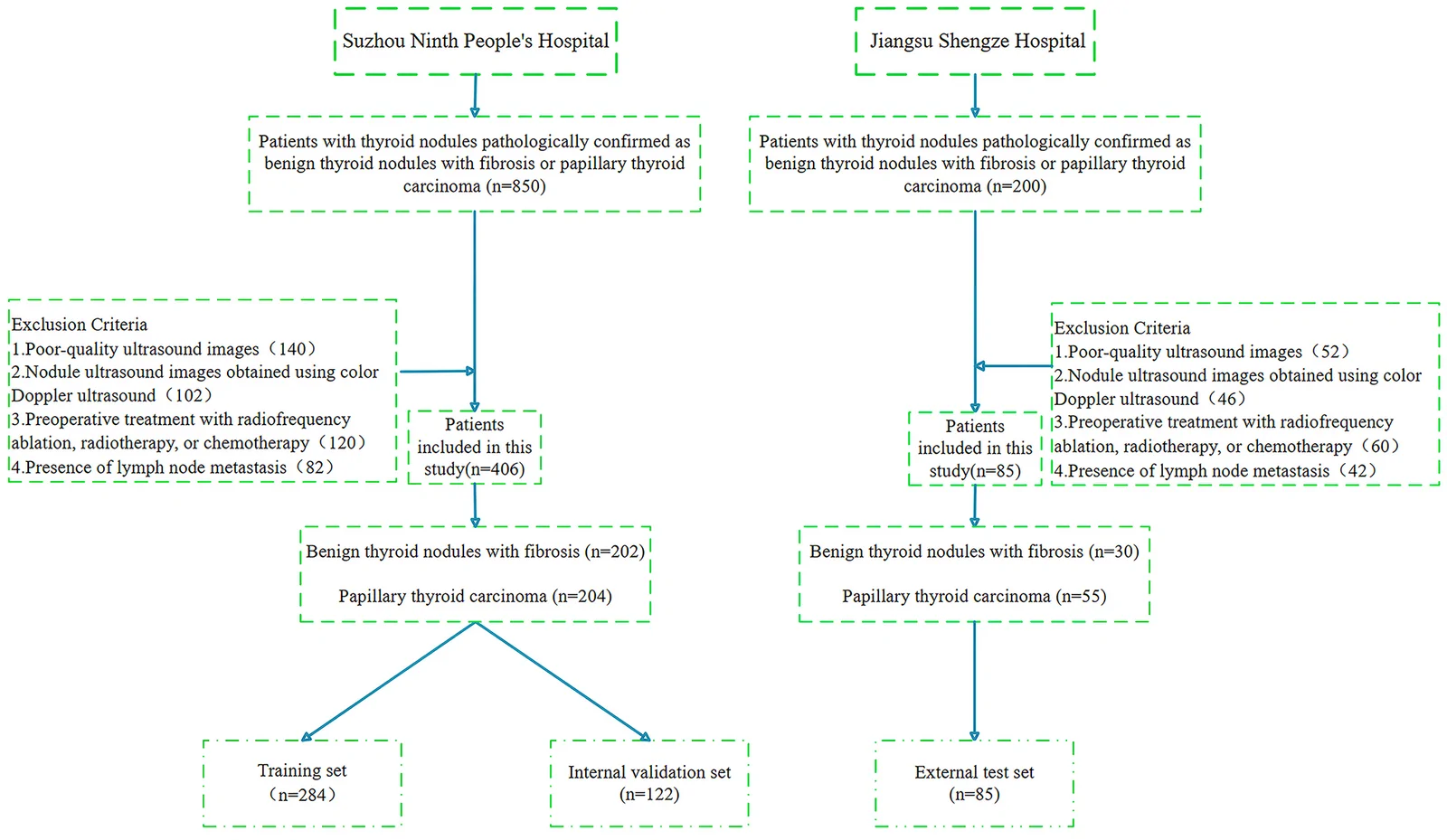
Patient inclusion and exclusion flowchart

This retrospective study included 491 patients with thyroid nodules from two hospitals. The inclusion criteria included: age between 18 and 75 years; preoperative thyroid ultrasound examination with TI-RADS grade ≥ 3; and confirmation of thyroid papillary carcinoma (PTC) or benign thyroid nodules with fibrotic changes after surgery. Exclusion criteria were: (1) poor ultrasound image quality (e.g., severe artifacts); (2) only color Doppler ultrasound images without grayscale images; (3) prior radiofrequency ablation, radiotherapy, or chemotherapy before surgery; (4) confirmed lymph node metastasis; (5) concurrent presence of other types of thyroid malignant tumors (e.g., follicular carcinoma, medullary carcinoma, undifferentiated carcinoma, etc.). Among these patients, Suzhou Ninth People’s Hospital included 406 cases (202 benign nodules with fibrotic changes and 204 PTC), and Jiangsu Shengze Hospital included 85 cases (30 benign nodules with fibrotic changes and 55 PTC). The enrollment process is shown in Fig. 1. Data from Suzhou Ninth People’s Hospital were divided into a training set (*n* = 284) and an internal validation set (*n* = 122) at a 7:3 ratio using stratified random sampling. This split was used for model development and hyperparameter tuning. Additionally, 85 patients from Jiangsu Jiangsu Shengze Hospital were selected as an independent external test set to evaluate the model’s cross-center generalization ability. This retrospective study was approved by the Ethics Committees of two hospitals, with anonymized patient identity information and a waiver of informed consent.

### Radiomics feature extraction and clinical data processing

#### Ultrasound image acquisition and preprocessing

The imaging equipment used in this study included the Toshiba Aplio 500 ultrasound system at the Ninth People’s Hospital of Suzhou (probe frequency 5–14 MHz) and the Philips EPIQ 7 ultrasound system at Jiangsu Shengze Hospital (probe frequency 5–12 MHz). All routine ultrasound examinations and image acquisitions were performed by attending ultrasound physicians with over 10 years of clinical experience to ensure baseline image quality.

To mitigate the domain shift stemming from disparities in texture artifacts and contrast characteristics between the Toshiba and Philips systems, a standardized image preprocessing pipeline was implemented prior to model training. First, original ultrasound images were manually cropped to isolate the Region of Interest (ROI) comprising the entire thyroid nodule, effectively eliminating peripheral vendor-specific graphical user interface (GUI) elements and calipers. Second, all cropped ROIs were resized to a uniform resolution of 224 × 224 pixels to align with the architectural input constraints of the neural network. Finally, pixel values were standardized using Z-score normalization to mitigate inter-vendor intensity heterogeneity. This workflow effectively minimized hardware-specific noise.

#### Radiomics feature extraction and selection

Two experienced ultrasound physicians, with 15 and 12 years of clinical practice respectively, independently evaluated the subjective radiological features (e.g., nodule size, morphology, margins) and outlined the regions of interest (ROI) using ITK-SNAP 3.8 software. To ensure the reliability of these assessments, inter-observer agreement was calculated, yielding a mean Cohen’s Kappa of 0.82 for categorical variables (e.g., margins, morphology) and an Intraclass Correlation Coefficient (ICC) of 0.88 for continuous variables (e.g., size), indicating excellent consistency.

In instances where ROI segmentation exhibited low labeling consistency (Dice coefficient < 0.85) or where disagreements arose regarding subjective feature assessment, a third senior physician with 20 years of experience arbitrated the discrepancies to reach a final consensus. After arbitration, the final ROI labeling consistency demonstrated a Dice coefficient of 0.89 ± 0.06. All finalized ROIs were meticulously delineated to encompass the complete nodule area while excluding surrounding normal tissue and blood vessels.

Subsequently, high-throughput radiomics features were extracted. Features with a proportion of missing values exceeding 5% and variance less than 1 × 10⁻⁶ were removed. Redundant features were further eliminated based on the Pearson correlation coefficient matrix (threshold > 0.95), ultimately retaining 1,282 robust features for subsequent analysis.

#### Clinical feature acquisition and preprocessing

To provide complementary semantic information and compensate for the limitations of imaging features alone, this study developed a structured clinical data branch. A total of nine key clinical features, including demographic information and ultrasound morphological characteristics, were retrospectively extracted from the hospital’s Electronic Medical Record (EMR) and Picture Archiving and Communication Systems (PACS).

Based on the ACR TI-RADS guidelines and routine clinical records, the input variables included: demographic factors (Age and Sex); pre-operative ultrasound features including maximum tumor diameter (Tumor Size), aspect ratio (< 1 or ≥ 1), nodule composition (solid, partially cystic, etc.), echogenicity (hyperechoic, isoechoic, hypoechoic, markedly hypoechoic), shape (regular, irregular), and calcification (none, microcalcification, coarse calcification); as well as a comprehensive risk score (Score) assessed by physicians according to TI-RADS standards. To ensure the objectivity of these clinical morphological features, the ultrasound reports and images were independently reviewed by two senior sonographers, each with over 10 years of experience in thyroid imaging, who were strictly blinded to the final surgical pathology results. In the event of discrepancies regarding specific morphological descriptions or TI-RADS scoring, a consensus was reached through adjudication by a third senior radiologist. Prior to model input, we performed Z-score normalization on the aforementioned data.

### Deep learning model development and training

In this study, we initialized 34 deep learning models with ImageNet pre-trained weights to evaluate their transfer learning performance on thyroid ultrasound tasks (Supplementary Table S1). We also compared the performance of different networks, including ResNet and DenseNet. Simultaneously, we extracted radiomics features from ultrasound images and combined them with clinical data including gender, age, and nodule size to construct four baseline models: Clinical, Rad, deep transfer learning (DTL), and deep learning radiomics (DLR). Using standard feature engineering strategies, radiomics features were selected by LASSO, and deep features were compressed to 512 dimensions.

**Fig. 2:**
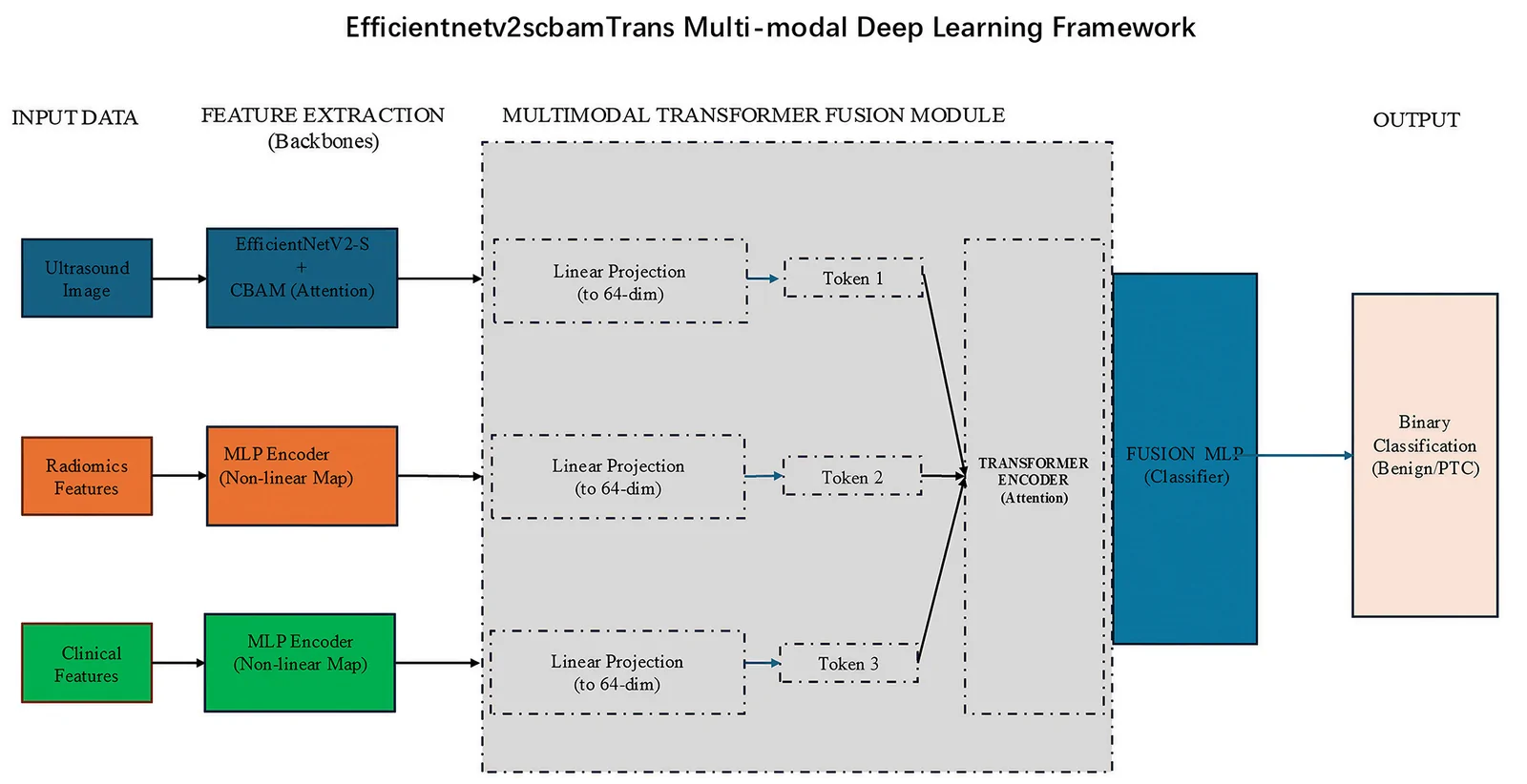
Architecture of the Efficientnetv2scbamTrans fusion model

On this basis, this study constructed an end-to-end multimodal fusion model, Efficientnetv2scbamTrans (see structural diagram in Fig. 2). This framework includes three feature extraction branches. The imaging branch uses the EfficientNetV2-S architecture as the backbone network and incorporates a convolutional block attention module (CBAM). This module enhances the feature response to key lesion areas such as microcalcifications. After global average pooling, a 256-dimensional imaging representation is generated. Concurrently, radiomics features selected by LASSO and raw clinical features (which were not subjected to feature selection) were encoded into high-level feature embeddings through modality-specific multi-layer perceptrons (MLPs), generating vector representations of dimensions 32 and 32, respectively.

Subsequently, a multimodal Transformer fusion module was introduced to calculate the correlation weights between different modalities. To effectively integrate the heterogeneous data, a dimensionality alignment step was first performed. Each modality’s output embedding was passed through a linear projection layer to map them into a unified latent space with a consistent dimension ($\:{d}_{model}=64$). These projected vectors were then concatenated to form a sequential input sequence, treating each modality as a distinct “token.” The Transformer module, primarily built upon Multi-Head Self-Attention (MHSA) mechanisms and Feed-Forward Networks (FFN), receives this sequence to dynamically compute cross-modal attention weights (e.g., evaluating how a specific microcalcification pattern correlates with a patient’s age or a texture feature). Layer Normalization and residual connections were applied to stabilize deep training. Finally, the contextually enriched token sequences were aggregated and fed into a classification head. This head is an MLP constructed with hidden units of [16, 32, 16], ultimately yielding the probability distribution for the binary classification task.

The model integrates Grad-CAM and SHAP techniques for visual analysis. To ensure the rigorous reproducibility of our study, the detailed training configurations were explicitly defined. The model was implemented using the PyTorch deep learning framework, and the global random seed was fixed at 42 to ensure reproducible evaluation. During training, the cross-entropy loss function was employed, and the network weights were updated using the AdamW optimizer. The training process was conducted for a maximum of 50 epochs with a batch size of 32. We set an initial learning rate of $\:3\times\:{10}^{-4}$ and a weight decay of $\:5\times\:{10}^{-4}$ along with L2 regularization to prevent overfitting. Furthermore, modality-specific dropout strategies were applied to enhance generalization: 0.2 for the vision branch, 0.5 for the radiomics branch, 0.3 for the clinical branch, and 0.1 within the Transformer fusion module. Data augmentation techniques were utilized, and TensorFloat-32 (TF32) with high-precision matrix multiplication were enabled to optimize computational efficiency. Strict adherence to data preprocessing and normalization standards was maintained, and all models underwent 5-fold cross-validation to ensure performance stability. A summary of the detailed training configurations is presented in Supplementary Table S2.

### Ablation study design

To systematically evaluate the contribution of each component, five ablation studies were designed. All experiments were conducted using the same data split, random seed (42), optimizer (AdamW), batch size (32), learning rate schedule, and early stopping strategy (patience = 15).The five experiments were: A (unimodal imaging baseline), B (imaging combined with clinical features), C (imaging combined with radiomics features), D (trimodal model without the CBAM), and E (full model). We analyzed the importance of each module by comparing metrics such as AUC and sensitivity. Additionally, we revealed cross-modal interaction mechanisms using SHAP methods.

### Model performance evaluation and interpretability analysis

Grad-CAM was used to generate model attention heatmaps for visual interpretability. To quantitatively evaluate the spatial alignment between the generated heatmaps and the manually delineated ROIs (tumor regions), three metrics were introduced: energy concentration index (EC), peak-centric distance (PCD), and spatial Intersection over Union (IoU). Notably, to avoid the bias of relying on a single arbitrary threshold for continuous heatmap binarization, we employed a robust multi-threshold averaging strategy for Spatial IoU calculation. Specifically, the raw Grad-CAM heatmaps were first Min-Max normalized to a continuous scale of [0, 1]. Subsequently, a sequence of progressive binarization thresholds ($\:T\in\:\left\{\mathrm{0.5,0.6,0.7,0.8,0.9}\right\}$) was applied. At each threshold * T*, pixels with values $\:\ge\:T$ were defined as the attention foreground, and an independent IoU was computed against the binary manual ROI. The final Spatial IoU metric for each sample was defined as the arithmetic mean of these multi-threshold IoUs. Based on DenseNet201 as the baseline, the improvement in each metric after introducing the convolutional block attention module (CBAM) was systematically compared. The area under the curve (AUC) and its 95% confidence interval were calculated using ROC curves, while sensitivity, specificity, accuracy, and additional classification metrics such as precision and F1-score were computed based on confusion matrices. The Hosmer-Lemeshow test was used to evaluate model calibration, and decision curve analysis (DCA) was employed to calculate clinical net benefit. DeLong test was used to compare AUC differences between models, while McNemar test was used for classification metrics comparison, both performed with Bonferroni correction. The generalization ability was evaluated by the AUC difference between the training set and the test set, where an AUC difference less than 0.05 was considered indicative of excellent generalization.

### Computational environment and statistical analysis

Normality of continuous variables was assessed using the Shapiro-Wilk test. For independent group comparisons (e.g., patient baseline characteristics between training and test sets), normally distributed variables were analyzed using the independent samples t-test, while non-normally distributed variables were analyzed using the Mann-Whitney U test. For paired comparisons of continuous model evaluation metrics on the same test set (specifically, the Grad-CAM attention quality metrics including EC, PCD, and Spatial IoU), the paired samples t-test was used for normally distributed data, whereas the non-parametric Wilcoxon signed-rank test was applied for non-normally distributed data. Categorical variables were analyzed using the chi-square test or Fisher’s exact test. As detailed in section “Model performance evaluation and interpretability analysis”, the DeLong test and McNemar test with Bonferroni correction were used for AUC and comparisons of classification metrics, respectively. All analyses were performed on the OnekeyAI platform (version 4.9.1) running in a Python 3.7.12 environment. Statistical evaluations were conducted using Statsmodels (version 0.13.2) and SciPy (version 1.7.3), radiomics features were extracted using PyRadiomics (version 3.0.1), machine learning was implemented with Scikit-learn (version 1.0.2), and deep learning was performed using PyTorch (version 1.11.0). To ensure full computational reproducibility, all deep learning model training and inference were conducted on a dedicated local workstation equipped with an NVIDIA GeForce RTX 4090 GPU with 24 GB of VRAM, and 64 GB of system RAM. CUDA 11.3.1 and cuDNN 8.2.1 were used to accelerate hardware computations. All statistical tests were two-sided, with a significance level α = 0.05.

## Results

### Baseline and clinical characteristics of patients and correlation analysis

This study included 491 patients with thyroid nodules. Baseline characteristics are shown in Table 1. The training set, validation set, and external test set have been relatively balanced in terms of age (*p* = 0.84), nodule composition (*p* = 0.54), and tumor size (*p* = 0.39), with no statistically significant differences. However, significant differences were found between the training set and the external test set in gender distribution, echogenicity, and horizontal diameter ratio (*p* < 0.001). In terms of pathological subtypes, 206 cases (79.5%) were classic PTC, 32 cases (12.4%) were follicular subtype, and 21 cases (8.1%) were high-cell variant. Among benign nodules with fibrosis, 148 cases (63.8%) were nodular goiter, 59 cases (25.4%) were adenomatous nodules, and 25 cases (10.8%) were Hashimoto’s thyroiditis-related nodules. In this study, univariate and multivariate logistic regression analyses based on training data (Table 2) revealed that tumor size (OR = 0.924, *p* < 0.001), risk score (OR = 1.130, *p* = 0.01), and specific morphological features (OR = 3.528, *p* = 0.02) were independent predictors for differentiating benign and malignant lesions. Considering their statistical significance and clinical relevance, these features were then incorporated into building the Clinical baseline model.

**Table 1:** Baseline characteristics across training, validation, and test sets

Feature Name	All	Test	Train	Validation	*p*-value
Tumor Size (Mean ± SD)	14.15 ± 11.52	15.59 ± 10.92	13.65 ± 11.41	14.30 ± 12.16	0.39
Score (Mean ± SD)	6.03 ± 2.18	5.51 ± 2.06	6.16 ± 2.20	6.12 ± 2.17	0.05
Age (Mean ± SD)	54.27 ± 12.51	54.98 ± 13.38	54.18 ± 12.35	53.96 ± 12.34	0.84
Composition n(%)					0.54
Cystic / Spongiform	3 (0.61)	1 (1.18)	1 (0.35)	1 (0.82)	
Cystic-solid	80 (16.29)	10 (11.76)	52 (18.31)	18 (14.75)	
Solid	408 (83.09)	74 (87.06)	231 (81.34)	103 (84.43)	
Echogenicity n(%)					< 0.001
High (Hyperechoic)	7 (1.43)	4 (4.71)	2 (0.70)	1 (0.82)	
Iso (Isoechoic)	395 (80.45)	4 (4.71)	277 (97.54)	114 (93.44)	
Low (Hypoechoic)	84 (17.11)	72 (84.7)	5 (1.76)	7 (5.74)	
Very Low	5 (1.01)	5 (5.88)	0 (0.00)	0 (0.00)	
Horizontal Diameter Ratio n(%)					< 0.001
< 1 (Wider than tall)	360 (73.32)	76 (89.41)	195 (68.66)	89 (72.95)	
≥ 1 (Taller than wide)	131 (26.68)	9 (10.59)	89 (31.34)	33 (27.05)	
Shape n(%)					0.05
Regular	432 (87.98)	74 (87.06)	254 (89.44)	104 (85.25)	
Irregular	50 (10.18)	7 (8.23)	29 (10.21)	14 (11.47)	
Other / Lobulated	9 (1.84)	4 (4.71)	1 (0.35)	4 (3.28)	
Calcification n(%)					0.01
None	227 (46.23)	48 (56.47)	128 (45.07)	51 (41.80)	
Macrocalcification	96 (19.55)	7 (8.24)	57 (20.07)	32 (26.23)	
Peripheral calcification	24 (4.89)	0 (0.00)	17 (5.99)	7 (5.74)	
Microcalcification	144 (29.33)	30 (35.29)	82 (28.87)	32 (26.23)	
Sex n(%)					< 0.001
Male	158 (32.18)	63 (74.12)	67 (23.59)	28 (22.95)	
Female	333 (67.82)	22 (25.88)	217 (76.41)	94 (77.05)	

**Table 2:** Univariate and multivariate logistic regression analysis

Feature Name	Univariate		Multivariate	
	OR (95%CI)	*P* value	OR (95%CI)	*P* value
Tumor Size	0.967(0.954–0.979)	**0.00**	0.924(0.901–0.947)	**0.00**
Echogenicity	0.980(0.810–1.186)	0.86		
Age	0.997(0.994–1.001)	0.18		
Sex	1.028(0.822–1.285)	0.84		
Score	1.040(1.009–1.071)	**0.03**	1.130(1.045–1.221)	**0.01**
Calcification	1.060(0.947–1.185)	0.40		
Composition	1.072(0.965–1.191)	0.28		
Horizontal Diameter Ratio	3.046(2.032–4.563)	**0.00**	1.549(0.803–2.986)	0.27
Shape	3.933(1.874–8.256)	**0.00**	3.528(1.461–8.525)	**0.02**

### Comparison of deep learning architectures

Among 34 ImageNet-pretrained architectures, DenseNet201 performed the best. It achieved a training set AUC of 0.862 and a test set AUC of 0.702, indicating moderate overfitting. ImageNet pretraining did not bring significant performance improvement, and some lightweight models (e.g., MobileNetV3-Small, AUC = 0.512) and Transformer architectures (SimpleViT, AUC = 0.537) performed poorly, indicating significant negative transfer. Overfitting was widespread, with ResNet101 showing a training-test AUC gap of 0.296. The ResNet series had moderate performance (ResNet50 AUC = 0.678). Based on these results, DenseNet201 was selected as the backbone network. However, its single-modal performance remained insufficient to meet clinical requirements (Supplementary Table 1).

### Comparison of traditional methods performance

The performance comparison results of the four traditional methods showed that the Clinical model had an AUC of 0.786, a sensitivity of 85.5%, and a specificity of 73.3%. The order of feature importance was as follows: echogenicity, morphology, and margin. The Rad model had an AUC of 0.765, a sensitivity of 80.0%, and a specificity of 76.7%. However, feature redundancy limited the discriminative value of individual features. The DTL model had an AUC of 0.739, a sensitivity of 61.8%, and a specificity of 83.3%. This performance was lower than that of the Clinical model, suggesting the presence of “negative transfer” in traditional fusion modes. In other words, model performance declined during fusion. The DLR model used an ExtraTrees classifier to fuse deep learning and radiomics features, achieving an AUC of 0.787. Compared with the Clinical model, the difference was not statistically significant (*p* = 0.92), indicating certain limitations in this two-stage fusion strategy. In summary, the Clinical model exhibited the best performance, the DTL model confirmed the occurrence of negative transfer, and the DLR model highlighted limitations of traditional fusion approaches (Fig. 2).

### End-to-end model result analysis

**Fig. 3:**
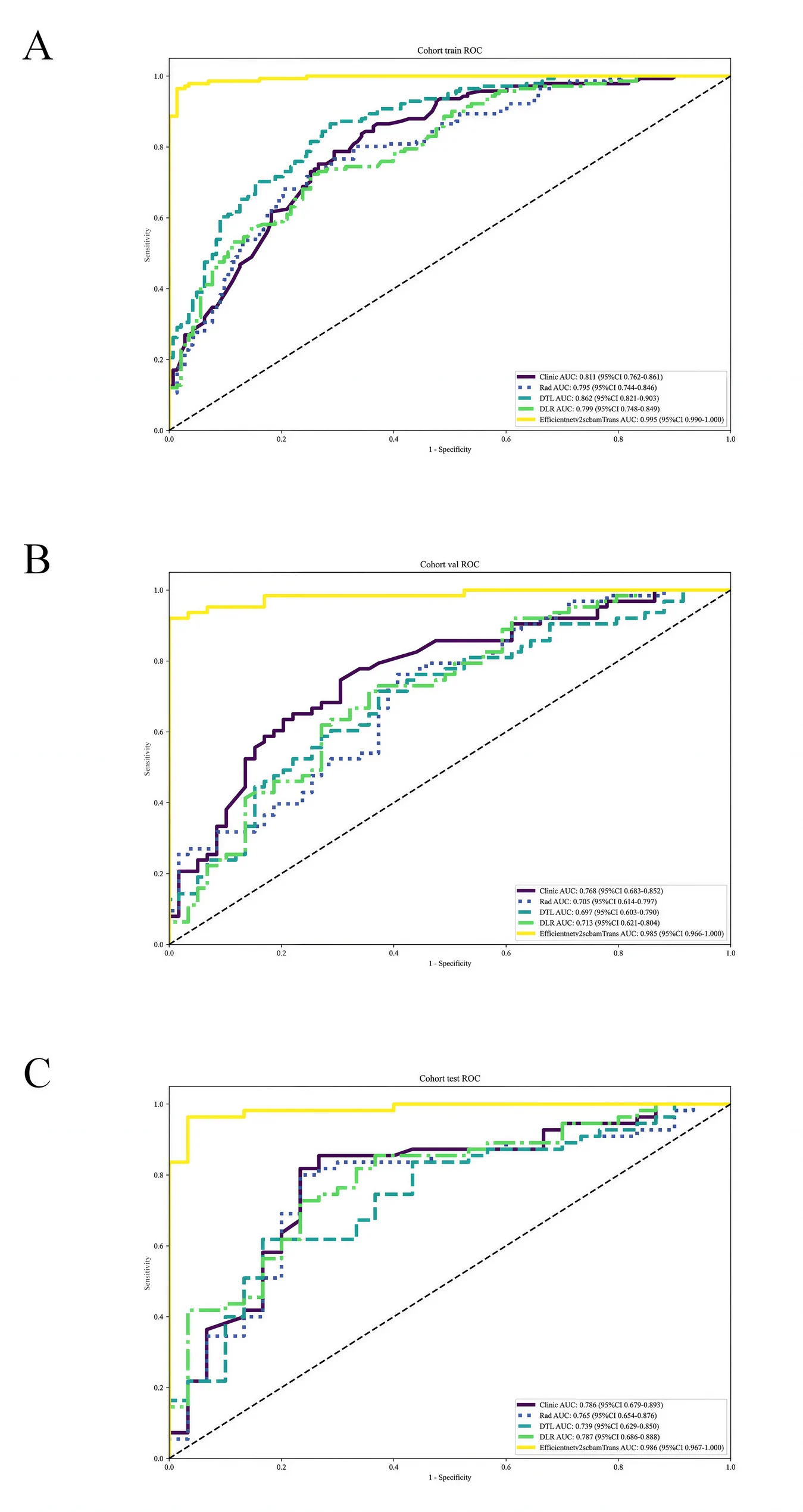
ROC curves comparing model performance across datasets

**Fig. 4:**
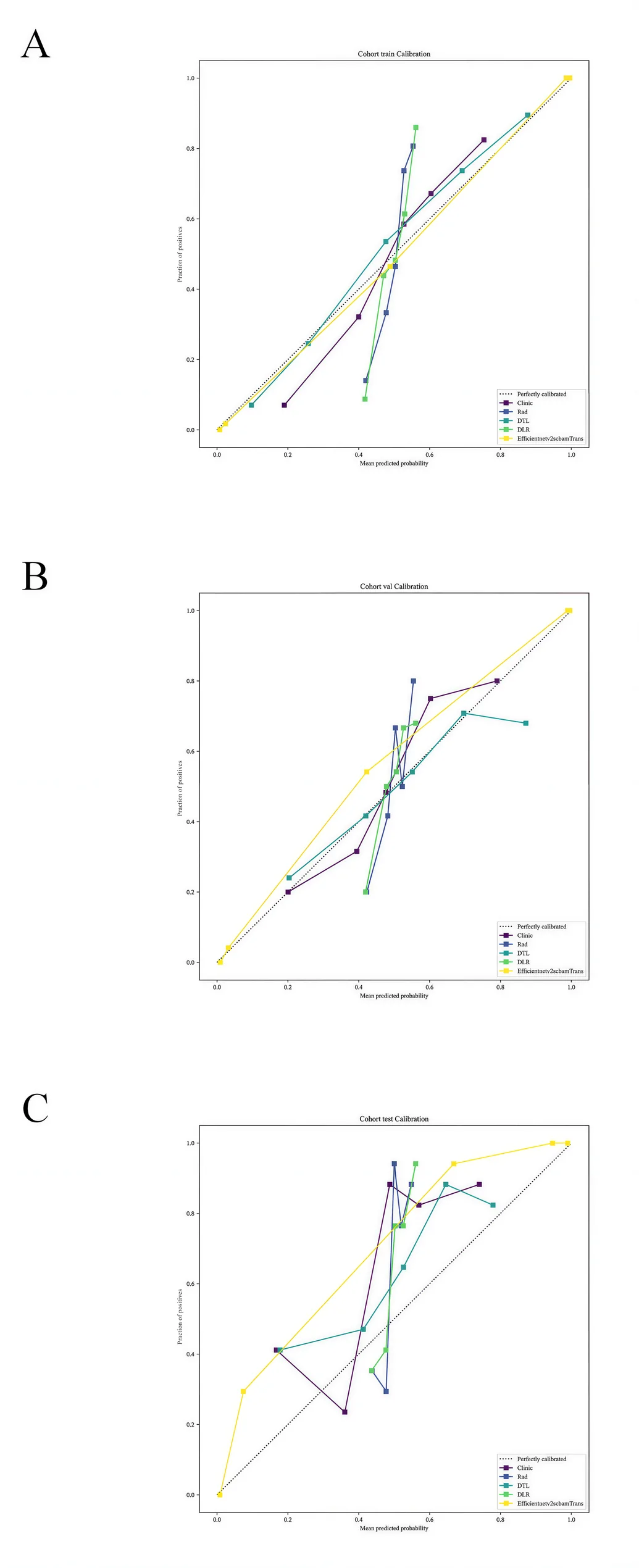
Grad-CAM visualization and attention quality metrics

**Fig. 5:**
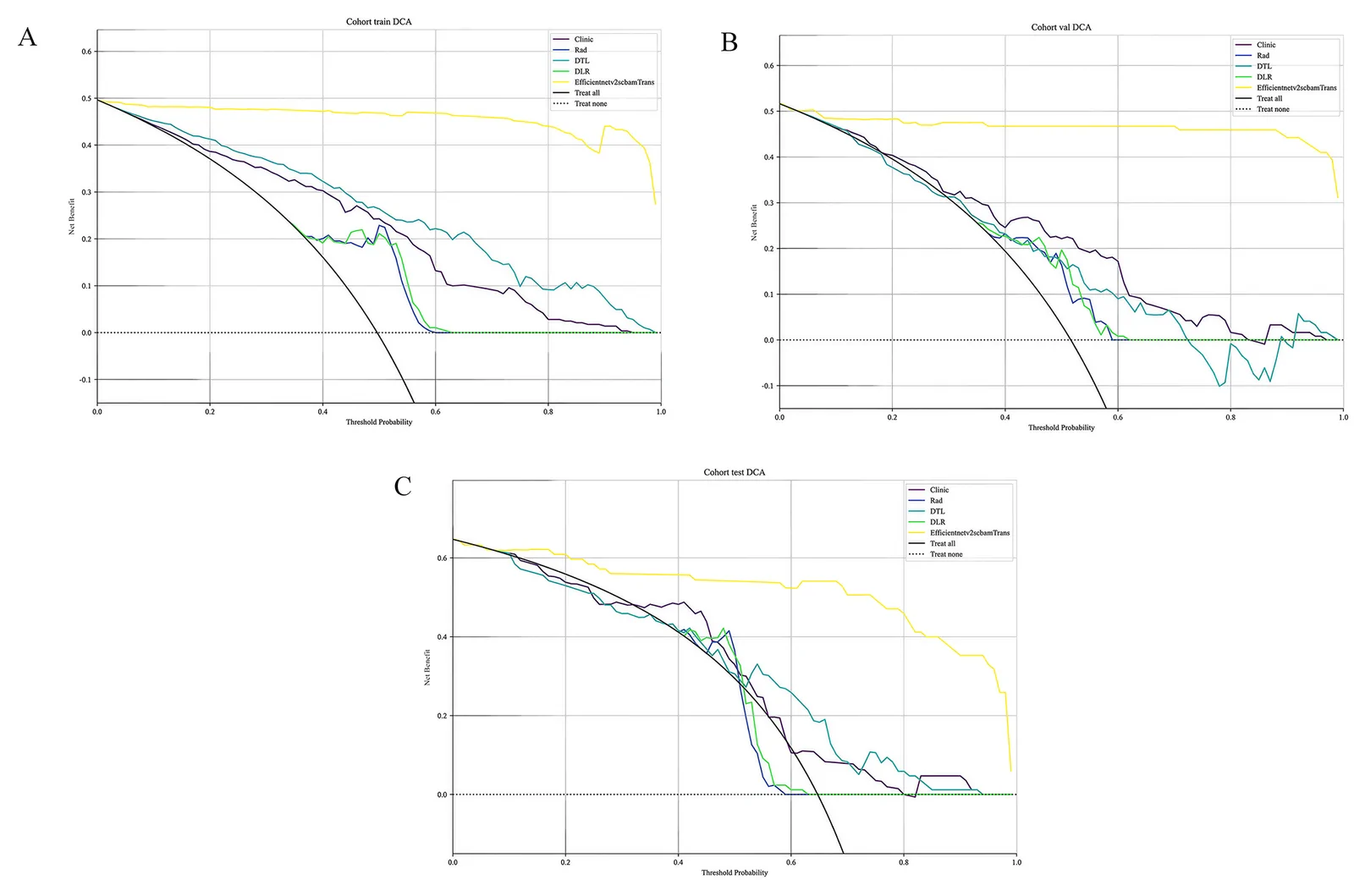
Subgroup attention analysis across nodule phenotypes

**Fig. 6:**
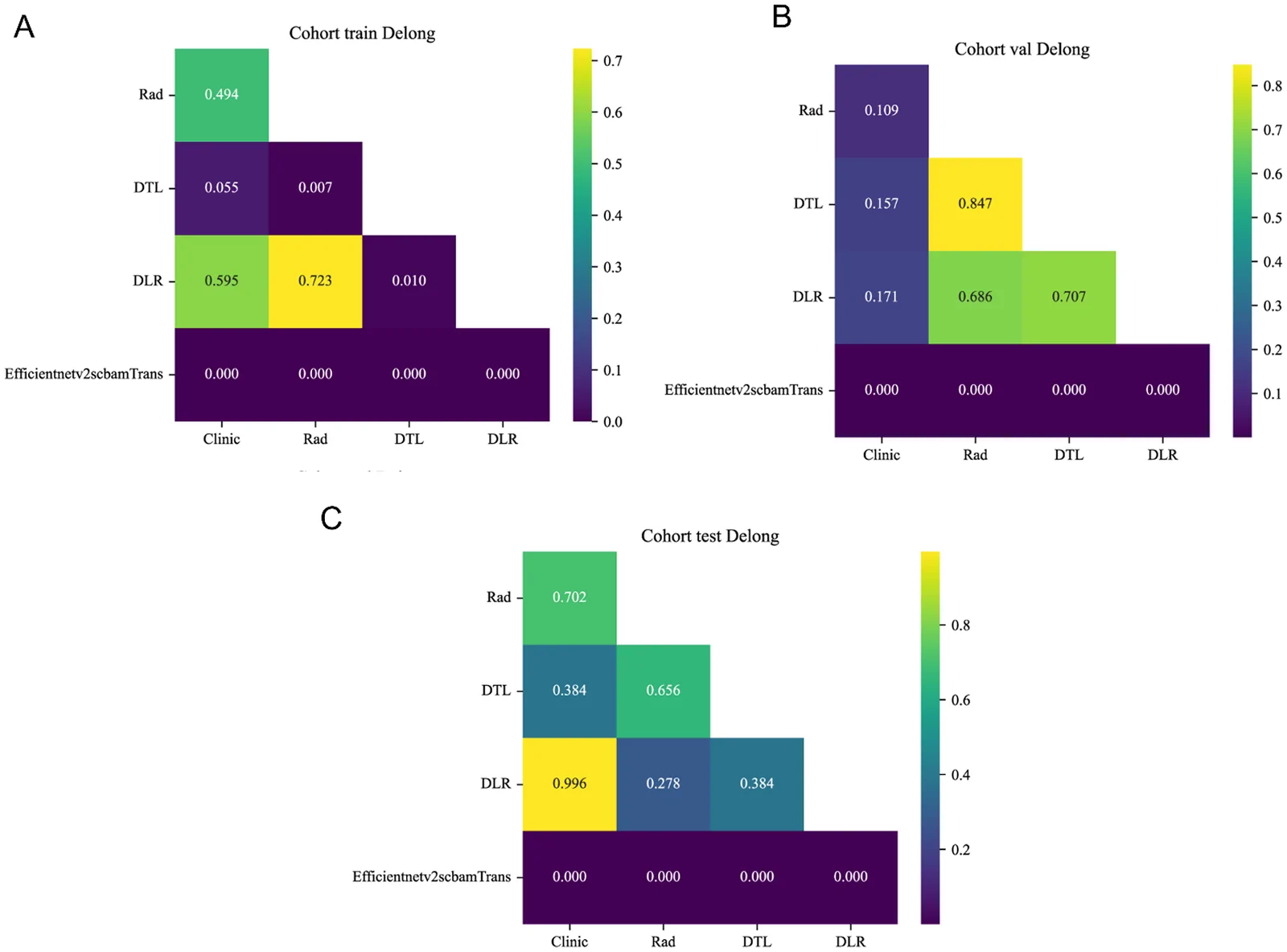
AUC heatmap across experiments and dataset splits

Efficientnetv2scbamTrans fusion model achieved an AUC of 0.986 on the test set (Fig. 3) and an accuracy of 96.5%. This performance significantly outperforms all traditional models: Clinical (81.2%), Rad (78.8%), DTL (69.4%), and DLR (74.1%) (Table 3). The model achieves a good balance between sensitivity (96.4%) and specificity (96.7%). In contrast, the Clinical model has a lower positive predictive value (PPV) of 73.3%, while the DTL model lacks specificity (61.8%) and exhibits a higher false positive rate compared to Efficientnetv2scbamTrans fusion model. Moreover, Efficientnetv2scbamTrans fusion model has higher positive predictive value (98.1%) and negative predictive value (NPV) (93.5%), indicating very low rates of misdiagnosis and missed diagnoses (Table 3). Calibration curves show high consistency between predicted probabilities and actual probabilities (Hosmer–Lemeshow χ²=2.87, *p* = 0.943)(Fig. 4). The decision curve analysis indicates that at a threshold probability of 0.5, Efficientnetv2scbamTrans fusion model has a net benefit of 0.39, significantly higher than the DLR (0.26, an improvement of 50%) and Clinical (0.28, an improvement of 39%) models (Fig. 5). DeLong test results (Fig. 6) show that Efficientnetv2scbamTrans fusion model has significantly higher AUC in the training set, validation set, and independent test set compared to the four traditional models; all comparison p-values are less than 0.001.

**Table 3:** Model performance comparison across cohorts

Signature	Accuracy	AUC	95% CI	Sensitivity	Specificity	PPV	NPV	Cohort
Clinic	0.746	0.811	0.762–0.861	0.861	0.865	0.629	0.697	train
Rad	0.739	0.795	0.744–0.846	0.846	0.681	0.797	0.768	train
DTL	0.789	0.862	0.821–0.903	0.903	0.865	0.713	0.748	train
DLR	0.732	0.799	0.748–0.849	0.849	0.730	0.734	0.730	train
Efficientnetv 2scbamTrans	0.975	0.995	0.990 -1.000	1	0.965	0.986	0.986	train
Clinic	0.721	0.768	0.683–0.852	0.852	0.746	0.695	0.723	val
Rad	0.68	0.705	0.614–0.797	0.797	0.762	0.593	0.667	val
DTL	0.672	0.697	0.603–0.790	0.790	0.714	0.627	0.672	val
DLR	0.68	0.713	0.621–0.804	0.804	0.714	0.644	0.682	val
Efficientnetv 2scbamTrans	0.959	0.985	0.966 -1.000	1	0.921	1	1	val
Clinic	0.812	0.786	0.679–0.893	0.893	0.855	0.733	0.855	test
Rad	0.788	0.765	0.654–0.876	0.876	0.800	0.767	0.863	test
DTL	0.694	0.739	0.629–0.849	0.849	0.618	0.833	0.872	test
DLR	0.741	0.787	0.686–0.888	0.727	0.767	0.851	0.605	test
Efficientnetv 2scbamTrans	0.965	0.986	0.967 -1.000	0.964	0.967	0.981	0.935	test

### Grad-CAM attention quality analysis

**Fig. 7:**
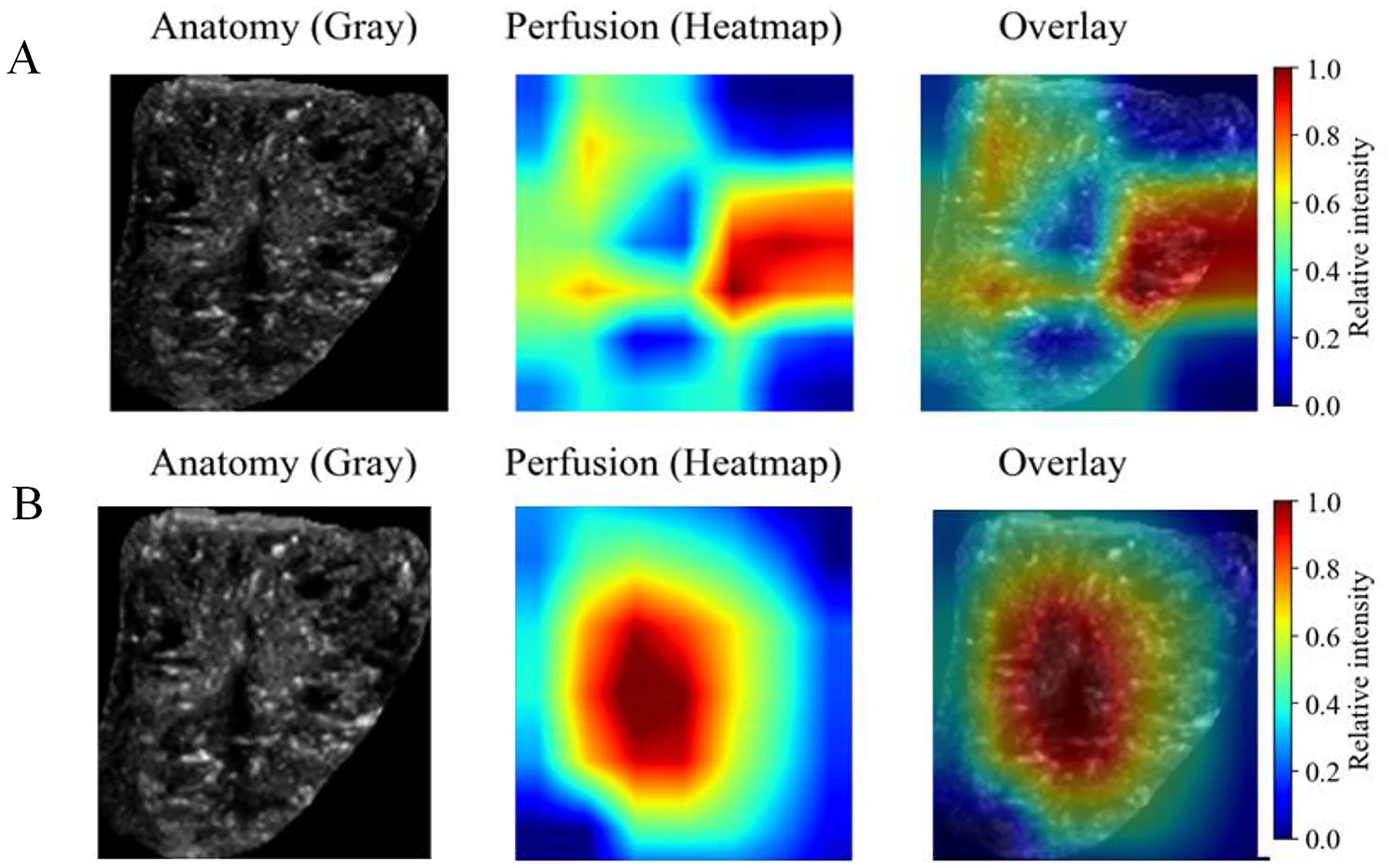
Radar chart of test set performance metrics

**Fig. 8:**
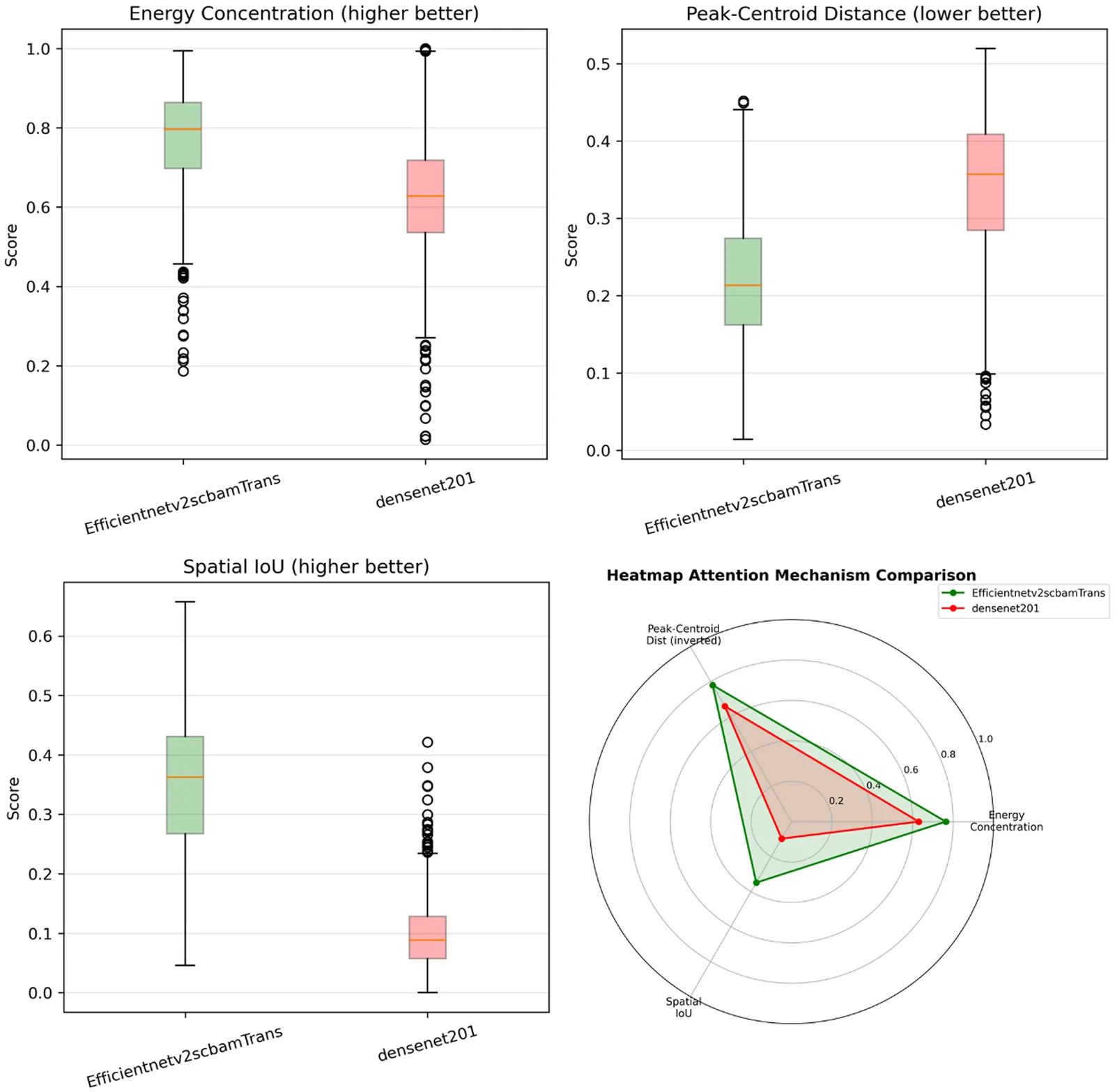
SHAP feature importance analysis

**Fig. 9:**
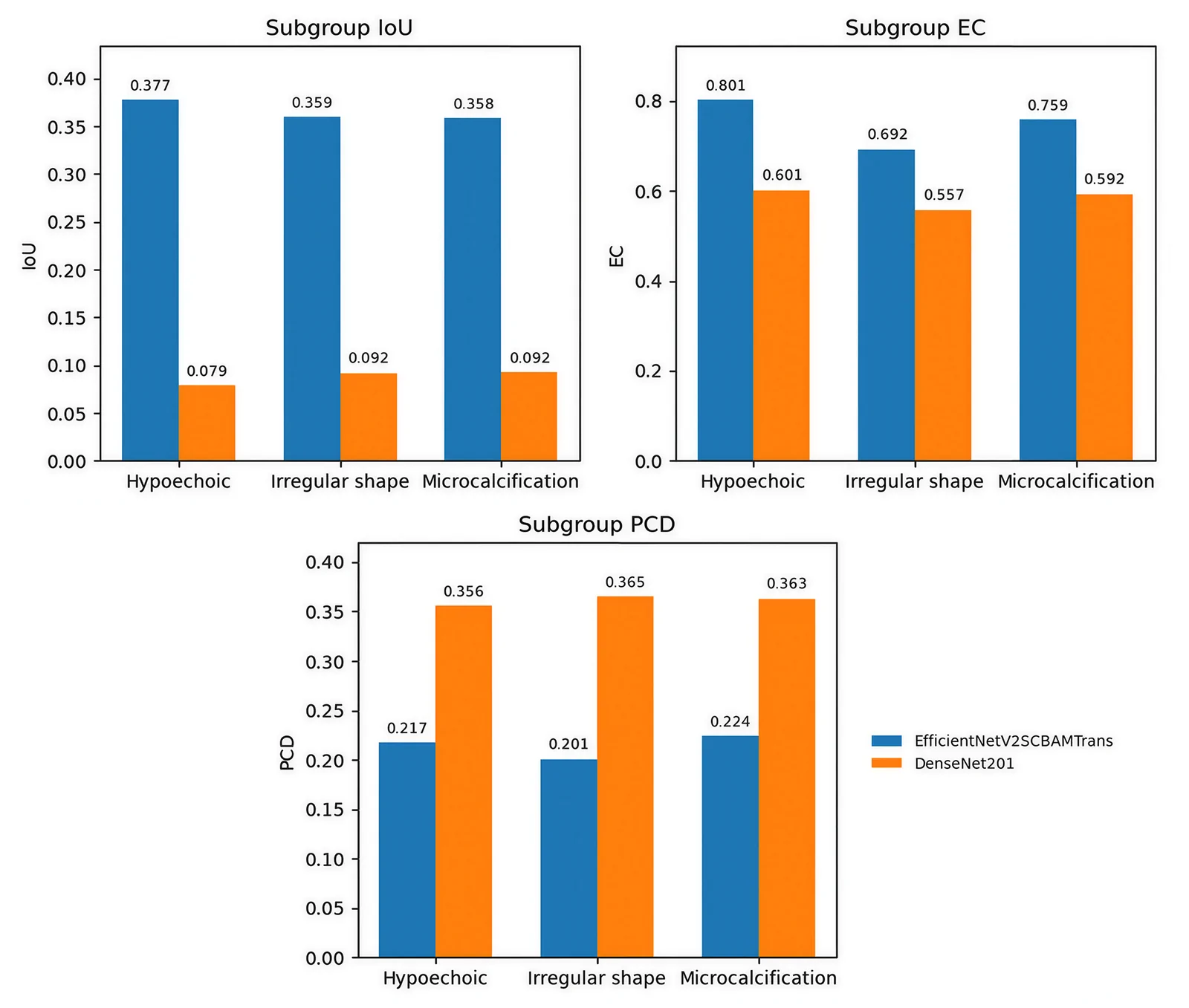
Attention heatmap comparison across nodule subtypes

Figure 7 shows the Grad-CAM visualization results for DenseNet201 (A) and Efficientnetv2scbamTrans fusion model (B). Compared to DenseNet201, Efficientnetv2scbamTrans fusion model exhibits more concentrated activation in lesion areas and a higher overlap with ground truth regions. This indicates that the model’s attention is more focused on key lesions. Quantitative analysis further reveals that Efficientnetv2scbamTrans fusion model has a significantly higher attention energy concentration (EC) (0.764 vs. 0.630, *p* = 1.1 × 10⁻³⁹), a significantly lower peak-to-centroid distance (PCD) (0.220 vs. 0.340, *p* = 3.6 × 10⁻⁵³), and a significantly higher spatial intersection over union (IoU) (0.349 vs. 0.097, *p* = 3.4 × 10⁻⁸¹). These results suggest that the CBAM module effectively enhances spatial focus and improves model interpretability (Fig. 8; Table 4). A further clinical feature stratified analysis (Fig. 9; Table 5) shows that Efficientnetv2scbamTrans fusion model significantly outperforms DenseNet201 in all subgroups. The attention distribution exhibits clear subtype-specific differences. In low-echo nodules, the IoU increased from 0.079 to 0.377, nearly a 5-fold improvement, and EC rose by 0.20 (from 0.601 to 0.801), indicating that the model has the strongest localization and focusing ability on these lesions. In irregular-shaped nodules, PCD decreased by 0.16 compared to DenseNet201, dropping from 0.365 to 0.201. This indicates that the attention peak aligns more closely with the lesion centroid. In microcalcification nodules, IoU and EC remained at high levels (0.358 and 0.759), while PCD was at an intermediate level (0.224), suggesting the model maintains stable spatial attention quality across various nodule morphologies. Therefore, the CBAM module can dynamically redistribute channel and spatial weights based on different ultrasound phenotypes, achieving a differentiated attention mechanism that aligns with clinical prior features. This feature-adaptive focusing mechanism significantly outperforms DenseNet201 in both localization accuracy and interpretability, providing quantitative support for the interpretability of multimodal fusion models.

**Table 4:** Attention quality metrics comparison

Metric	Efficientnetv2scbamTrans	DenseNet201	*p*-value
Energy Concentration (EC)	0.764	0.630	1.11E-39
Peak–Centroid Distance (normalized, PCD)	0.220	0.340	3.65 E-53
Spatial Intersection-over-Union (IoU, multi-threshold avg)	0.349	0.097	3.40 E-81

**Table 5:** Attention metrics by ultrasound phenotype subgroups

Feature	*n*	IoU (EffNetV2-SCBAMTrans)	EC (EffNetV2-SCBAMTrans)	PCD (EffNetV2-SCBAMTrans)	IoU (DenseNet201)	EC (DenseNet201)	PCD (DenseNet201)
Hypoechoic	84	0.377	0.801	0.217	0.079	0.601	0.356
Irregular shape	50	0.359	0.692	0.201	0.092	0.557	0.365
Microcalcification	144	0.358	0.759	0.224	0.092	0.592	0.363

### Ablation experiment results

**Fig. 10:**
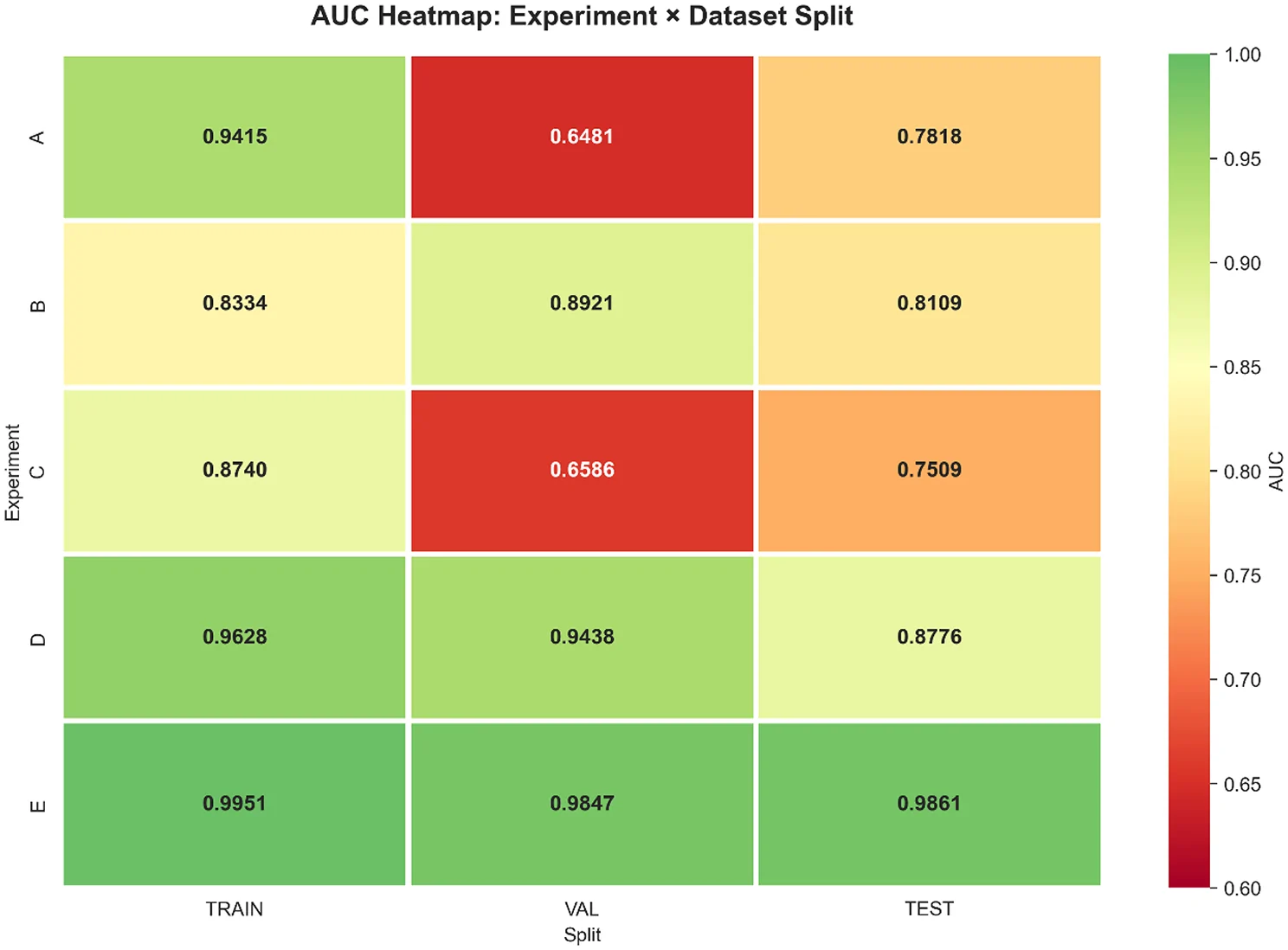
Ablation experiment results across five model configurations

**Fig. 11:**
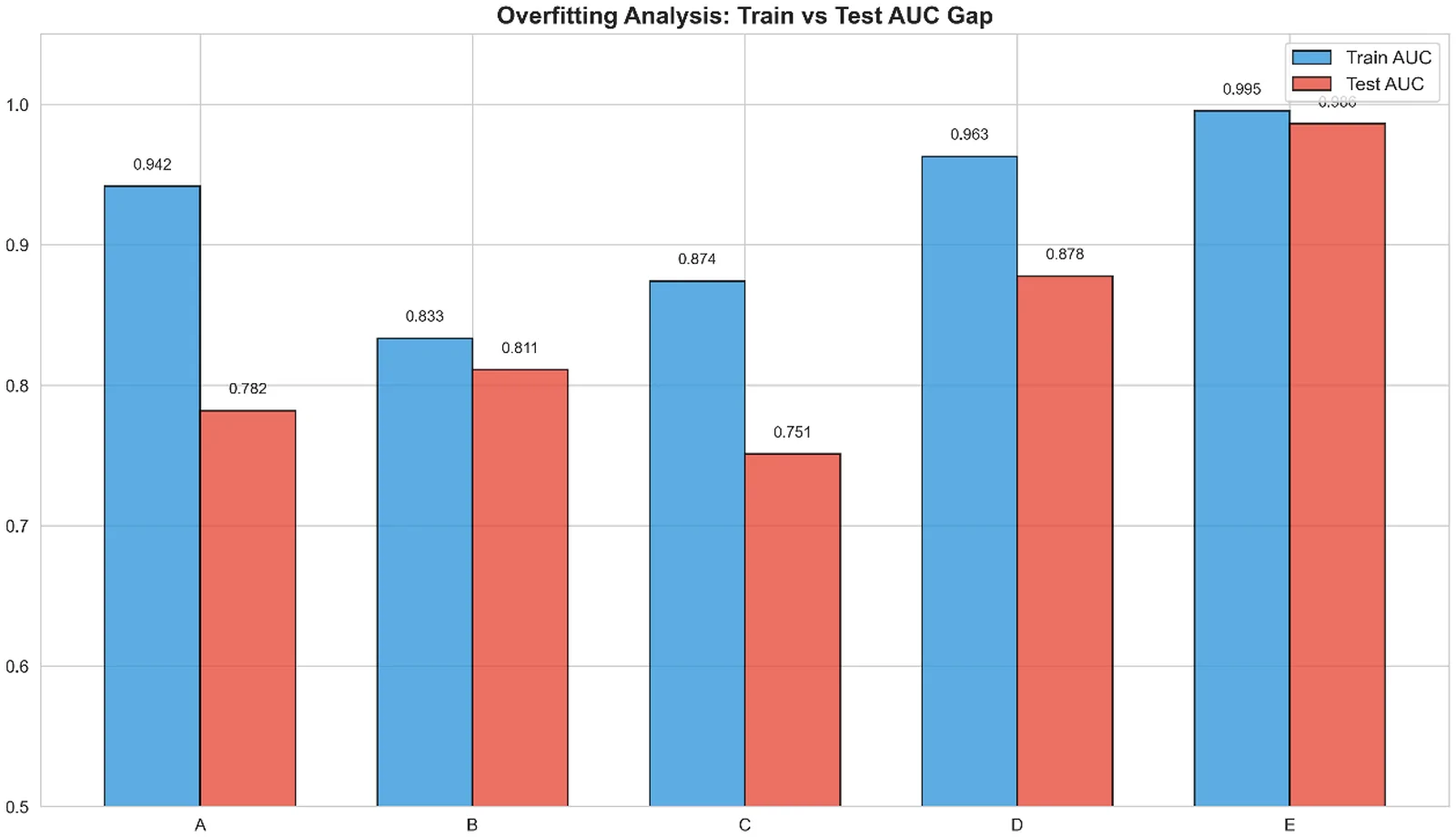
Generalization analysis: Train vs. Test AUC gap

**Fig. 12:**
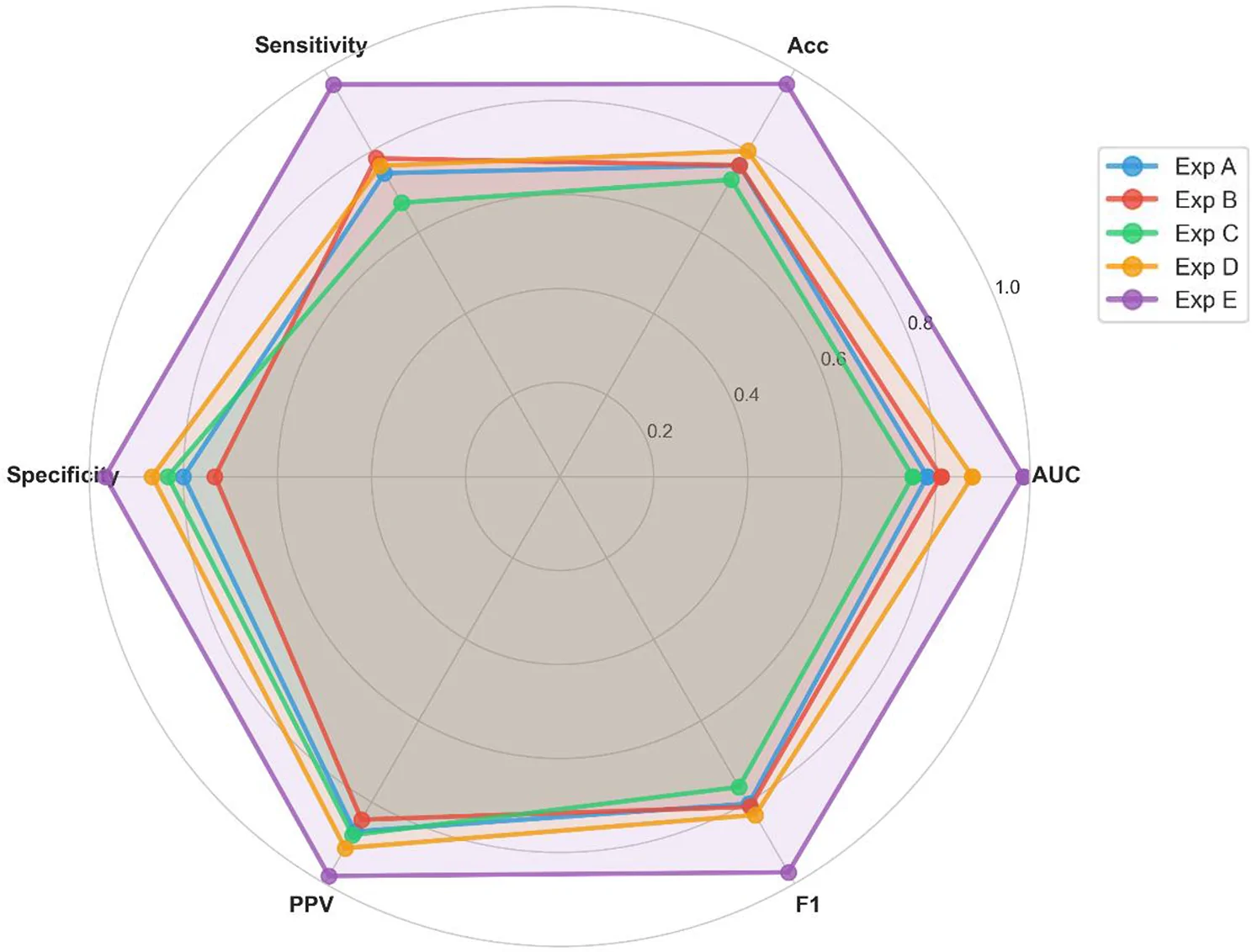
Component contribution summary

The specific performance metrics of the five ablation experiments are detailed in Table 6. Figure 10 visually presents the AUC distributions of the five experiments on the training, validation, and test sets using a heatmap. Figures 11 and 12 show the differences between training and testing under various experimental settings, along with radar chart analyses. The single-modal imaging baseline (Experiment A) achieved an AUC of 0.942 on the training set and 0.782 on the external test set. This significant gap of 0.160 clearly indicates model overfitting to the internal training data. A detailed comparison between Ablation Experiment B (imaging + clinical) and Experiment C (imaging + radiomics) revealed contrasting behavioral patterns when facing domain shifts (from internal validation to external testing). After incorporating clinical features (Experiment B), the training set AUC decreased to 0.833; however, the test set AUC improved to 0.811, and the train-test performance gap was drastically reduced to merely 0.022. This empirically demonstrates that clinical features (such as age, gender, and nodule size) serve as robust, objective prior knowledge, effectively acting as a regularizer to mitigate overfitting. In sharp contrast, Experiment C (imaging + radiomics) achieved an AUC of only 0.751 on the test set, which is even lower than the single-modal baseline (0.782). This abnormal phenomenon suggests that when using a simple concatenation strategy under cross-center domain shifts, high-dimensional radiomics features conflict with deep visual features. Instead of providing complementary value, they introduce redundant noise and mismatched information, thereby causing a negative transfer effect (i.e., model performance degradation). Experiment D (attention mechanism and multimodal synergy without CBAM) achieved an AUC of 0.878 on the test set.The complete model E (Efficientnetv2scbamTrans fusion model) achieved a significant leap in performance by introducing CBAM and Transformer fusion modules. This model achieved an AUC as high as 0.985 on the internal validation set. Even on an external test set with a significantly different data distribution, the model still maintained an excellent AUC of 0.986. The high consistency between internal validation and external testing strongly suggests that the Transformer’s self-attention mechanism effectively resolves feature conflicts and selects universal pathological features. Additionally, the complete model achieved the best balance in sensitivity (96.4%) and specificity (96.7%), demonstrating strong cross-center generalization ability and potential clinical applicability.

**Table 6:** Ablation experiment results

Experiment	Split	N_samples	Acc	AUC	CI	Sensitivity	Specificity	PPV	NPV	F1
A	TRAIN	284	0.8873	0.9415	0.9146–0.9654	0.9291	0.8462	0.8562	0.9237	0.8912
A	VAL	122	0.6639	0.6481	0.5452-0.7500	0.7937	0.5254	0.641	0.7045	0.7092
A	TEST	85	0.7647	0.7818	0.6717–0.8816	0.7455	0.8	0.8723	0.6316	0.8039
B	TRAIN	284	0.7852	0.8334	0.7830–0.8822	0.7021	0.8671	0.839	0.747	0.7645
B	VAL	122	0.8361	0.8921	0.8328–0.9447	0.873	0.7966	0.8209	0.8545	0.8462
B	TEST	85	0.7647	0.8109	0.7042–0.9021	0.7818	0.7333	0.8431	0.6471	0.8113
C	TRAIN	284	0.8134	0.874	0.8308–0.9115	0.844	0.7832	0.7933	0.8358	0.8179
C	VAL	122	0.6311	0.6586	0.5557–0.7541	0.5397	0.7288	0.68	0.5972	0.6018
C	TEST	85	0.7294	0.7509	0.6404–0.8539	0.6727	0.8333	0.881	0.5814	0.7629
D	TRAIN	284	0.9085	0.9628	0.9422–0.9796	0.8723	0.9441	0.9389	0.8824	0.9044
D	VAL	122	0.8689	0.9438	0.9038–0.9754	0.8571	0.8814	0.8852	0.8525	0.871
D	TEST	85	0.8	0.8776	0.7879–0.9499	0.7636	0.8667	0.913	0.6667	0.8317
E	TRAIN	284	0.9754	0.9951	0.9887–0.9990	0.9645	0.986	0.9855	0.9658	0.9749
E	VAL	122	0.959	0.9847	0.9642–0.9992	0.9206	1	1	0.9219	0.9587
E	TEST	85	0.9647	0.9861	0.9633-1.0000	0.9636	0.9667	0.9815	0.9355	0.9725

### SHAP explainability and cross-modal synergy analysis

**Fig. 13:**
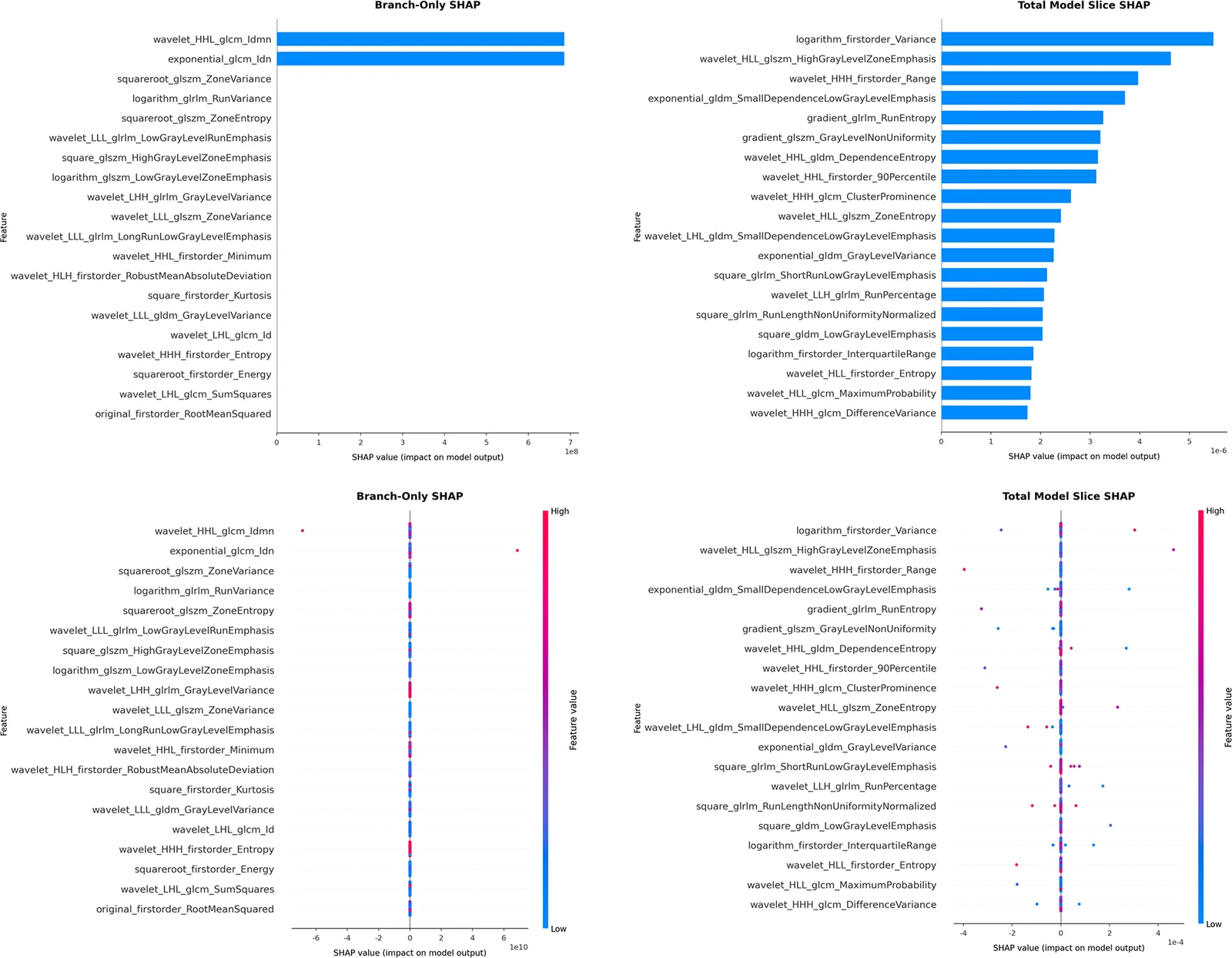
Cross-modal feature reshaping via SHAP analysis

The results of the ablation experiments highlight the importance of feature interaction mechanisms, especially the performance discrepancy between experiments C and E. To better understand why the complete model effectively utilizes radiomics features without being affected by noise, we analyzed feature contributions using the SHAP method. The model integrates approximately 1300-dimensional features and evaluates their importance using the average absolute SHAP value. In the modality-level analysis, the total SHAP contributions of radiomics and clinical features were 56.5% (Total_SHAP = 1.4067) and 43.5% (Total_SHAP = 1.0842), respectively (Table 7). Although clinical features comprise only 9 items, their average SHAP value (1.20 × 10⁻³) is significantly higher than that of radiomics (1.10 × 10⁻⁵), indicating that high-density clinical information plays an anchoring role in decision-making. We compared the feature rankings between the “radiomics-only branch” and the “radiomics subset in the fused model.” After fusion, the Gini coefficient increased from 0.0008 to 0.2546 (overall model Gini = 0.2109), indicating that the model concentrated on a few key features during the fusion process. The Spearman correlation between the SHAP distributions of the radiomics-only branch and the radiomics subset in the fused model was not statistically significant (ρ = 0.0177, *p* = 0.53), suggesting that fusion led to a significant feature reshuffling (Table 8). Some radiomics features showed a significant rank improvement after fusion (e.g., logarithm_glcm_Idn, wavelet_HHL_glcm_Idmn). These features mainly represent texture complexity and heterogeneity—patterns that complement clinical variables effectively. Conversely, some single-modal high-weight features, such as wavelet_LLH_glszm_SmallAreaEmphasis, ranked lower after fusion, indicating that their information was partially compensated for by clinical features (Fig. 13). This selective enhancement and suppression mechanism shows that clinical priors dynamically regulate the importance of radiomics features during fusion. By achieving information focus and feature reconstruction, this mechanism becomes key to improving model performance and generalization ability.

**Table 7:** SHAP-based feature contribution analysis

Type	N_Features	Mean_SHAP	Total_SHAP	SHAP_Ratio
RAD	1282	1.10-E5	1.4067	56.47
Clinical	9	0.001205	1.0842	43.53

**Table 8:** Feature importance redistribution in fusion model

Metric	Single-Modality RAD Branch Only	Fusion Model RAD Slice	Overall Fusion Model (Total Summary)
Gini Coefficient	0.0008	0.2546	0.2109
Spearman Correlation (ρ)	—	0.0177	—
p-value	—	0.53	—

## Discussion

This study systematically evaluated 34 deep learning architectures and various fusion strategies. It specifically explored the challenges these methods face in achieving cross-center generalization for thyroid ultrasound diagnosis. Although previous research has confirmed the potential of deep learning in uncovering deep patterns in images, this study found that directly transferring ImageNet pre-trained models—referred to here as Direct Transfer Learning (DTL)—or adopting traditional two-stage fusion strategies, known as Deep Learning-based Representation (DLR), perform poorly when dealing with heterogeneous data (external test set AUC < 0.80). The single-modal imaging baseline (experiment A) showed a significant performance drop from the internal training set (AUC 0.942) to the external test set (AUC 0.782). This result strongly validates the core challenges identified by the study’s two-center data design. Specifically, the significant domain shift stems from differences in device parameters and patient population characteristics. To address this challenge, this study proposed an end-to-end multi-modal fusion model named Efficientnetv2scbamTrans fusion model. It combines a lightweight backbone network (EfficientNetV2-S), a convolutional block attention module (CBAM), and a Transformer-based multi-modal fusion module, achieving an outstanding AUC of 0.986 (98.6%) on an independent external test set. The model’s cross-center robustness results from the combined advantages of its lightweight backbone architecture, the Transformer-driven multi-modal fusion mechanism, and effective strategies to suppress feature conflicts. Consequently, the model demonstrates strong generalization ability and stability.

Ultrasound examination is currently the primary method for evaluating thyroid nodules. According to the American College of Radiology Thyroid Imaging Reporting and Data System (ACR TI-RADS), ultrasound features of thyroid nodules such as margins, echo, and microcalcifications are used to assess their malignant risk [20]. Studies have shown that radiomic features can capture subtle structural differences that traditional imaging methods fail to identify, thereby providing more precise information for differentiating benign and malignant tumors [21]. Based on this, researchers use deep learning to analyze ultrasound images and extract detailed radiomics features that traditional imaging may overlook. This significantly improves the prediction of nodule malignancy risk [22]. Specifically, researchers have trained deep learning models to automatically classify and assess the risk of thyroid nodules, significantly enhancing diagnostic accuracy and efficiency [23]. Moreover, combining radiomics features with clinical variables—such as patient age, gender, and thyroid hormone levels—can further improve the accuracy of thyroid nodule risk assessment [24]. For example, some research results indicate that the proportion of female patients with thyroid cancer is as high as 60%, which may be related to the physiological effects of estrogen [25]. Therefore, incorporating patient age and gender into the assessment process can improve the accuracy of overall thyroid nodule evaluation [26]. Nevertheless, numerous challenges remain in combining radiomic features and clinical variables, which urgently require attention and resolution. First, the extraction and analysis process of radiomic features is complex. It involves the selection of various algorithms and models, which may affect the reproducibility and stability of results [27].The clinical application prospects of radiomic features remain broad; however, to realize their widespread use in daily medical practice, existing technical and methodological challenges must be overcome. With continuous technological advancements and in-depth research, radiomics is expected to play an increasingly important role in personalized medicine and precision medicine, providing more effective treatment plans and management strategies for patients.

This study demonstrates that backbone network selection is crucial for the generalization ability of small-sample medical data models. Although the model structure based on DenseNet201 is complex. However, it exhibits overfitting on the external test set. This may be due to the deep network’s tendency to memorize noise in the training data. In contrast, EfficientNetV2-S is a lightweight convolutional neural network that efficiently extracts pathological texture features through its Fused-MBConv structure and progressive learning strategy [28]. It also reduces the number of parameters. As Zhang et al. [29] pointed out, in medical image tasks with limited annotations, lightweight and targeted-optimized network architectures often avoid the common overfitting problems of deep networks. More importantly, the contrasting behaviors observed in our ablation studies—specifically between Experiment B (imaging + clinical) and Experiment C (imaging + radiomics)—provide crucial insights into multimodal fusion dynamics and strongly support our concluding claims. Although radiomics features theoretically can extract microscopic textures [30], naive concatenation of these features in multi-center research often leads to “negative transfer,” as evidenced by the performance decline in Experiment C (AUC dropping to 0.751). The fundamental reason is that unfiltered, high-dimensional radiomics features are exquisitely sensitive to imaging parameters such as probe frequency, angle, and gain. Zwanenburg et al. [31] confirmed that minor variations in image acquisition conditions can cause significant shifts in radiomics features. Consequently, when the model encounters data from different hospitals (domain shift), these rigid radiomics features carry substantial non-pathological “device fingerprint” noise, which degrades generalization capability. Conversely, Experiment B demonstrated that integrating clinical prior features successfully mitigated the overfitting observed in the baseline model (reducing the train-test gap to 0.022, achieving an external AUC of 0.811). Clinical variables in our study—encompassing objective demographic data (e.g., age, gender) and strictly controlled morphological priors (e.g., nodule composition and echogenicity, which were rigorously evaluated and arbitrated by blinded senior experts)—act as highly reliable features. Unlike high-dimensional radiomics, which are highly sensitive to hardware variations, these demographic metrics and standardized clinical priors remain largely independent of ultrasound hardware. By mapping these stable priors into the multimodal semantic space, they act as a strong regularization mechanism. They effectively anchor the learning process, preventing the deep learning network from overfitting to machine-specific imaging artifacts. However, traditional feature combination strategies still struggle to achieve optimal diagnostic accuracy. Therefore, the complete model Efficientnetv2scbamTrans fusion model was designed to overcome these exact limitations, boosting the AUC to 0.986. It demonstrates the profound advantage of the Transformer architecture in multi-modal information fusion. Finally, the model exhibits 96.7% specificity and 96.4% sensitivity on the external test set, performing excellently, partly due to architectural improvements and strict inclusion/exclusion criteria. These criteria excluded cases with poor image quality, which ensured training stability but also lowered the classification difficulty of the test set. Overall, these results should be considered the performance ceiling under high-quality ultrasound image conditions, with the model significantly improving specificity while enhancing sensitivity, showing clinical potential as a pre-FNA screening tool [32].

This study also has several limitations that need to be addressed in future work. First, despite using a dual-center design, the total sample size (491 cases) is relatively small, which may have prevented the Transformer-based deep network from fully learning the comprehensive feature distribution. Second, the study included only cases confirmed by surgery, excluding a large number of nodules that underwent fine-needle aspiration (FNA) follow-up or conservative observation; this may introduce selection bias and potentially reduce the model’s performance in screening the general clinical population. Third, only two mainstream devices, Toshiba and Philips, were evaluated, while other brands (e.g., GE, Siemens, Mindray) still require further validation. Fourth, the model did not integrate molecular markers such as BRAF and TERT, limiting its ability to achieve precise stratification. Finally, while the clinical and morphological features (such as TI-RADS descriptors) utilized in our multimodal network were strictly extracted and arbitrated by senior sonographers blinded to pathology to ensure high baseline data quality, this rigorous multi-reader consensus process is difficult to replicate in routine, high-throughput clinical practice. The inherent inter-observer variability among physicians with varying levels of experience in assessing ultrasound morphological features might introduce fluctuations in the clinical branch’s input, potentially affecting the model’s performance when deployed in standard, unarbitrated clinical workflows.

## Conclusion

This study uses a two-center design, showing that radiomics feature concatenation adds noise under domain shifts, while clinical prior features reduce overfitting. The Efficientnetv2scbamTrans fusion model effectively resolves multimodal conflicts and noise, demonstrating strong generalization on external test sets. Despite limitations in sample size and design, it offers an interpretable AI solution to minimize overdiagnosis of thyroid nodules, with future validation expected to enhance clinical decision-making.

## Supplementary Information

Below is the link to the electronic supplementary material.

Supplementary Material 1

Supplementary Material 2

## Data Availability

The data used in this study contain protected health information and are not publicly available due to ethical restrictions. However, to ensure reproducibility, the code for feature extraction, selection, and model building has been deposited in a public GitHub repository: https://github.com/minxu02/Application-of-Transformer-Attention-Mechanism-Based-Multimodal-Deep-Learning-Model.
