# Supplementary material for: Application of transformer attention mechanism-based multimodal deep learning model in the diagnosis of papillary thyroid carcinoma
Source: BMC Med Imaging. 2026 Jun 27;26:450. doi: 10.1186/s12880-026-02530-w (PMC13576103; doi:10.1186/s12880-026-02530-w)
Supplement: Supplementary file 1 [file 12880_2026_2530_MOESM1_ESM.docx]

| Supplementary Table S1. Performance of 34 ImageNet-Pretrained Models | | | | | | | | | | | | |
| --- | --- | --- | --- | --- | --- | --- | --- | --- | --- | --- | --- | --- |
| ModelName | Cohort | Acc | AUC | CI | Sensitivity | Specificity | PPV | NPV | Precision | Recall | F1 | Threshold |
| alexnet | Train | 0.704 | 0.742 | 0.6853-0.7992 | 0.773 | 0.636 | 0.677 | 0.74 | 0.677 | 0.773 | 0.722 | 0.457 |
| alexnet | Test | 0.643 | 0.646 | 0.5694-0.7224 | 0.712 | 0.551 | 0.677 | 0.59 | 0.677 | 0.712 | 0.694 | 0.511 |
| densenet121 | Train | 0.701 | 0.755 | 0.6997-0.8106 | 0.66 | 0.741 | 0.715 | 0.688 | 0.715 | 0.66 | 0.686 | 0.529 |
| densenet121 | Test | 0.691 | 0.695 | 0.6232-0.7677 | 0.856 | 0.472 | 0.682 | 0.712 | 0.682 | 0.856 | 0.759 | 0.515 |
| densenet161 | Train | 0.831 | 0.897 | 0.8618-0.9327 | 0.879 | 0.783 | 0.8 | 0.868 | 0.8 | 0.879 | 0.838 | 0.437 |
| densenet161 | Test | 0.647 | 0.665 | 0.5884-0.7410 | 0.661 | 0.629 | 0.703 | 0.583 | 0.703 | 0.661 | 0.681 | 0.756 |
| densenet169 | Train | 0.771 | 0.829 | 0.7814-0.8760 | 0.858 | 0.685 | 0.729 | 0.831 | 0.729 | 0.858 | 0.788 | 0.405 |
| densenet169 | Test | 0.575 | 0.597 | 0.5190-0.6752 | 0.542 | 0.618 | 0.653 | 0.505 | 0.653 | 0.542 | 0.593 | 0.72 |
| densenet201 | Train | 0.789 | 0.862 | 0.8206-0.9034 | 0.865 | 0.713 | 0.748 | 0.843 | 0.748 | 0.865 | 0.803 | 0.388 |
| densenet201 | Test | 0.667 | 0.702 | 0.6296-0.7736 | 0.653 | 0.685 | 0.733 | 0.598 | 0.733 | 0.653 | 0.691 | 0.532 |
| googlenet | Train | 0.725 | 0.785 | 0.7316-0.8380 | 0.709 | 0.741 | 0.73 | 0.721 | 0.73 | 0.709 | 0.719 | 0.521 |
| googlenet | Test | 0.647 | 0.643 | 0.5675-0.7193 | 0.856 | 0.371 | 0.643 | 0.66 | 0.643 | 0.856 | 0.735 | 0.494 |
| inception_v3 | Train | 0.623 | 0.647 | 0.5834-0.7111 | 0.582 | 0.664 | 0.631 | 0.617 | 0.631 | 0.582 | 0.605 | 0.586 |
| inception_v3 | Test | 0.599 | 0.629 | 0.5527-0.7055 | 0.5 | 0.73 | 0.711 | 0.524 | 0.711 | 0.5 | 0.587 | 0.76 |
| mnasnet0_5 | Train | 0.532 | 0.504 | 0.4365-0.5710 | 0.816 | 0.252 | 0.518 | 0.581 | 0.518 | 0.816 | 0.634 | 0.013 |
| mnasnet0_5 | Test | 0.609 | 0.523 | 0.4409-0.6043 | 0.924 | 0.191 | 0.602 | 0.654 | 0.602 | 0.924 | 0.729 | 0.001 |
| mnasnet1_0 | Train | 0.581 | 0.587 | 0.5236-0.6507 | 0.574 | 0.587 | 0.579 | 0.583 | 0.579 | 0.574 | 0.577 | 0.962 |
| mnasnet1_0 | Test | 0.618 | 0.563 | 0.5210-0.6040 | 0.966 | 0.157 | 0.603 | 0.778 | 0.603 | 0.966 | 0.743 | 1 |
| mobilenet_v2 | Train | 0.87 | 0.929 | 0.8998-0.9589 | 0.872 | 0.867 | 0.866 | 0.873 | 0.866 | 0.872 | 0.869 | 0.494 |
| mobilenet_v2 | Test | 0.671 | 0.669 | 0.5933-0.7440 | 0.729 | 0.596 | 0.705 | 0.624 | 0.705 | 0.729 | 0.717 | 0.488 |
| mobilenet_v3_large | Train | 0.673 | 0.715 | 0.6552-0.7744 | 0.745 | 0.601 | 0.648 | 0.705 | 0.648 | 0.745 | 0.693 | 0.484 |
| mobilenet_v3_large | Test | 0.628 | 0.655 | 0.5797-0.7304 | 0.729 | 0.494 | 0.656 | 0.579 | 0.656 | 0.729 | 0.691 | 0.378 |
| mobilenet_v3_small | Train | 0.644 | 0.681 | 0.6192-0.7428 | 0.709 | 0.58 | 0.625 | 0.669 | 0.625 | 0.709 | 0.664 | 0.503 |
| mobilenet_v3_small | Test | 0.628 | 0.631 | 0.5552-0.7064 | 0.619 | 0.64 | 0.695 | 0.559 | 0.695 | 0.619 | 0.655 | 0.499 |
| resnet101 | Train | 0.789 | 0.866 | 0.8241-0.9071 | 0.837 | 0.741 | 0.761 | 0.822 | 0.761 | 0.837 | 0.797 | 0.459 |
| resnet101 | Test | 0.671 | 0.656 | 0.5795-0.7329 | 0.788 | 0.517 | 0.684 | 0.648 | 0.684 | 0.788 | 0.732 | 0.664 |
| resnet152 | Train | 0.532 | 0.449 | 0.3819-0.5164 | 0.993 | 0.077 | 0.515 | 0.917 | 0.515 | 0.993 | 0.678 | 0.033 |
| resnet152 | Test | 0.614 | 0.638 | 0.5613-0.7142 | 0.661 | 0.551 | 0.661 | 0.551 | 0.661 | 0.661 | 0.661 | 0.619 |
| resnet18 | Train | 0.567 | 0.567 | 0.4999-0.6335 | 0.34 | 0.79 | 0.615 | 0.549 | 0.615 | 0.34 | 0.438 | 0.842 |
| resnet18 | Test | 0.652 | 0.607 | 0.5282-0.6861 | 0.754 | 0.517 | 0.674 | 0.613 | 0.674 | 0.754 | 0.712 | 0.483 |
| resnet34 | Train | 0.785 | 0.851 | 0.8084-0.8945 | 0.851 | 0.72 | 0.75 | 0.831 | 0.75 | 0.851 | 0.797 | 0.458 |
| resnet34 | Test | 0.676 | 0.675 | 0.6004-0.7488 | 0.881 | 0.404 | 0.662 | 0.72 | 0.662 | 0.881 | 0.756 | 0.331 |
| resnet50 | Train | 0.877 | 0.95 | 0.9271-0.9724 | 0.901 | 0.853 | 0.858 | 0.897 | 0.858 | 0.901 | 0.879 | 0.437 |
| resnet50 | Test | 0.643 | 0.627 | 0.5497-0.7046 | 0.644 | 0.64 | 0.704 | 0.576 | 0.704 | 0.644 | 0.673 | 0.491 |
| resnext101_32x8d | Train | 0.933 | 0.977 | 0.9633-0.9909 | 0.929 | 0.937 | 0.936 | 0.931 | 0.936 | 0.929 | 0.932 | 0.55 |
| resnext101_32x8d | Test | 0.671 | 0.681 | 0.6064-0.7548 | 0.746 | 0.573 | 0.698 | 0.63 | 0.698 | 0.746 | 0.721 | 0.55 |
| resnext50_32x4d | Train | 0.849 | 0.917 | 0.8857-0.9490 | 0.894 | 0.804 | 0.818 | 0.885 | 0.818 | 0.894 | 0.854 | 0.42 |
| resnext50_32x4d | Test | 0.643 | 0.669 | 0.5948-0.7436 | 0.551 | 0.764 | 0.756 | 0.562 | 0.756 | 0.551 | 0.637 | 0.669 |
| shufflenet_v2_x0_5 | Train | 0.595 | 0.603 | 0.5377-0.6691 | 0.759 | 0.434 | 0.569 | 0.646 | 0.569 | 0.759 | 0.65 | 0.5 |
| shufflenet_v2_x0_5 | Test | 0.522 | 0.532 | 0.4532-0.6103 | 0.28 | 0.843 | 0.702 | 0.469 | 0.702 | 0.28 | 0.4 | 0.501 |
| shufflenet_v2_x1_0 | Train | 0.496 | 0.465 | 0.3980-0.5319 | 1 | 0 | 0.496 | 0 | 0.496 | 1 | 0.664 | 0.49 |
| shufflenet_v2_x1_0 | Test | 0.502 | 0.489 | 0.4103-0.5681 | 0.322 | 0.742 | 0.623 | 0.452 | 0.623 | 0.322 | 0.425 | 0.503 |
| SimpleViT | Train | 0.553 | 0.533 | 0.4656-0.6004 | 0.489 | 0.615 | 0.556 | 0.55 | 0.556 | 0.489 | 0.521 | 0.503 |
| SimpleViT | Test | 0.517 | 0.537 | 0.4574-0.6163 | 0.271 | 0.843 | 0.696 | 0.466 | 0.696 | 0.271 | 0.39 | 0.566 |
| squeezenet1_0 | Train | 0.676 | 0.728 | 0.6704-0.7862 | 0.801 | 0.552 | 0.638 | 0.738 | 0.638 | 0.801 | 0.711 | 0.436 |
| squeezenet1_0 | Test | 0.565 | 0.599 | 0.5212-0.6761 | 0.449 | 0.719 | 0.679 | 0.496 | 0.679 | 0.449 | 0.541 | 0.595 |
| squeezenet1_1 | Train | 0.775 | 0.848 | 0.8044-0.8909 | 0.78 | 0.769 | 0.769 | 0.78 | 0.769 | 0.78 | 0.775 | 0.553 |
| squeezenet1_1 | Test | 0.667 | 0.643 | 0.5648-0.7205 | 0.797 | 0.494 | 0.676 | 0.647 | 0.676 | 0.797 | 0.732 | 0.504 |
| vgg11 | Train | 0.599 | 0.63 | 0.5662-0.6947 | 0.291 | 0.902 | 0.745 | 0.563 | 0.745 | 0.291 | 0.418 | 0.517 |
| vgg11 | Test | 0.681 | 0.716 | 0.6451-0.7871 | 0.644 | 0.73 | 0.76 | 0.607 | 0.76 | 0.644 | 0.697 | 0.509 |
| vgg11_bn | Train | 0.651 | 0.677 | 0.6148-0.7387 | 0.702 | 0.601 | 0.635 | 0.672 | 0.635 | 0.702 | 0.667 | 0.513 |
| vgg11_bn | Test | 0.657 | 0.663 | 0.5880-0.7374 | 0.729 | 0.562 | 0.688 | 0.61 | 0.688 | 0.729 | 0.708 | 0.5 |
| vgg13 | Train | 0.736 | 0.811 | 0.7612-0.8598 | 0.78 | 0.692 | 0.714 | 0.762 | 0.714 | 0.78 | 0.746 | 0.495 |
| vgg13 | Test | 0.681 | 0.668 | 0.5922-0.7443 | 0.831 | 0.483 | 0.681 | 0.683 | 0.681 | 0.831 | 0.748 | 0.499 |
| vgg13_bn | Train | 0.694 | 0.737 | 0.6795-0.7954 | 0.872 | 0.517 | 0.641 | 0.804 | 0.641 | 0.872 | 0.739 | 0.45 |
| vgg13_bn | Test | 0.667 | 0.691 | 0.6190-0.7637 | 0.661 | 0.674 | 0.729 | 0.6 | 0.729 | 0.661 | 0.693 | 0.5 |
| vgg16 | Train | 0.754 | 0.825 | 0.7778-0.8726 | 0.844 | 0.664 | 0.713 | 0.812 | 0.713 | 0.844 | 0.773 | 0.406 |
| vgg16 | Test | 0.662 | 0.67 | 0.5951-0.7450 | 0.695 | 0.618 | 0.707 | 0.604 | 0.707 | 0.695 | 0.701 | 0.513 |
| vgg16_bn | Train | 0.68 | 0.727 | 0.6693-0.7850 | 0.702 | 0.657 | 0.669 | 0.691 | 0.669 | 0.702 | 0.685 | 0.515 |
| vgg16_bn | Test | 0.633 | 0.671 | 0.5963-0.7447 | 0.602 | 0.674 | 0.71 | 0.561 | 0.71 | 0.602 | 0.651 | 0.518 |
| vgg19 | Train | 0.732 | 0.81 | 0.7608-0.8585 | 0.837 | 0.629 | 0.69 | 0.796 | 0.69 | 0.837 | 0.756 | 0.411 |
| vgg19 | Test | 0.681 | 0.666 | 0.5894-0.7426 | 0.856 | 0.449 | 0.673 | 0.702 | 0.673 | 0.856 | 0.754 | 0.394 |
| vgg19_bn | Train | 0.697 | 0.736 | 0.6788-0.7940 | 0.894 | 0.503 | 0.64 | 0.828 | 0.64 | 0.894 | 0.746 | 0.451 |
| vgg19_bn | Test | 0.628 | 0.663 | 0.5878-0.7374 | 0.525 | 0.764 | 0.747 | 0.548 | 0.747 | 0.525 | 0.617 | 0.501 |
| wide_resnet101_2 | Train | 0.634 | 0.632 | 0.5671-0.6969 | 0.489 | 0.776 | 0.683 | 0.607 | 0.683 | 0.489 | 0.57 | 0.562 |
| wide_resnet101_2 | Test | 0.614 | 0.635 | 0.5594-0.7105 | 0.568 | 0.674 | 0.698 | 0.541 | 0.698 | 0.568 | 0.626 | 0.502 |
| wide_resnet50_2 | Train | 0.708 | 0.754 | 0.6973-0.8112 | 0.645 | 0.769 | 0.734 | 0.687 | 0.734 | 0.645 | 0.687 | 0.567 |
| wide_resnet50_2 | Test | 0.623 | 0.621 | 0.5442-0.6976 | 0.593 | 0.663 | 0.7 | 0.551 | 0.7 | 0.593 | 0.642 | 0.492 |
