# Supplementary material for: Application of transformer attention mechanism-based multimodal deep learning model in the diagnosis of papillary thyroid carcinoma
Source: BMC Med Imaging. 2026 Jun 27;26:450. doi: 10.1186/s12880-026-02530-w (PMC13576103; doi:10.1186/s12880-026-02530-w)
Supplement: Supplementary file 2 [file 12880_2026_2530_MOESM2_ESM.docx]

Supplementary Table S2. Training Configurations and Hyperparameters of the Proposed Model

| Configuration Parameter | Value / Strategy |
| --- | --- |
| Deep Learning Framework | PyTorch |
| Global Random Seed | 42 |
| Optimizer | AdamW |
| Initial Learning Rate | 3×10⁻⁴ |
| Weight Decay | 5×10⁻⁴ |
| Batch Size | 32 |
| Maximum Epochs | 50 |
| Loss Function | Cross-Entropy Loss |
| Dimensionality Alignment (d_model) | 64 |
| Dropout (Vision / Rad / Clinical / Fusion) | 0.2 / 0.5 / 0.3 / 0.1 |
| Classification Head (Hidden Units) | [16, 32, 16] |
| Hardware Acceleration | cuDNN Benchmark, TF32 enabled |
